# Neurodevelopment of HIV-exposed uninfected children in Cape Town, South Africa

**DOI:** 10.1371/journal.pone.0242244

**Published:** 2020-11-18

**Authors:** Hlengiwe P. Madlala, Landon Myer, Thokozile R. Malaba, Marie-Louise Newell

**Affiliations:** 1 Division of Epidemiology and Biostatistics, School of Public Health and Family Medicine, University of Cape Town, Cape Town, Western Cape, South Africa; 2 Centre for Infectious Disease Epidemiology and Research, School of Public Health and Family Medicine, University of Cape Town, Cape Town, Western Cape, South Africa; 3 School of Human Development and Health, Faculty of Medicine, University of Southampton, Southampton, United Kingdom; 4 School of Public Health, Faculty of Health Sciences, University of Witwatersrand, Johannesburg, South Africa; University of Botswana, BOTSWANA

## Abstract

**Background:**

Evidence shows that antiretroviral (ART) exposure is associated with neurodevelopmental delays in human immunodeficiency virus (HIV)-exposed uninfected (HEU) children. However, there are few insights into modifiable maternal and child factors that may play a role in improving neurodevelopment in HEU children. We used a parent-centric neurodevelopment tool, Ages & Stages Questionnaire (ASQ) to examined neurodevelopment in HEU children at 12–24 months of age, and associations with maternal and child factors.

**Methods:**

505 HIV-infected women (initiated ART pre- or during pregnancy) with live singleton births attending primary health care were enrolled; 355 of their HEU children were assessed for neurodevelopment (gross motor, fine motor, communication, problem solving and personal-social domains) at 12–24 months using age-specific ASQ administered by a trained fieldworker. Associations with maternal and child factors were examined using logistic regression models.

**Results:**

Among mothers (median age 30 years, IQR, 26–34), 52% initiated ART during pregnancy; the median CD4 count was 436 cells/μl (IQR, 305–604). Most delayed neurodevelopment in HEU children was in gross (9%) and fine motor (5%) functions. In adjusted models, maternal socio-economic status (aOR 0.42, 95% CI 0.24–0.76) was associated with reduced odds of delayed gross-fine motor neurodevelopment. Maternal age ≥35 years (aOR 0.22, 95% CI 0.05–0.89) and maternal body mass index (BMI) <18.5 (aOR 6.76, 95% CI 1.06–43.13) were associated with delayed communication-problem-solving-personal-social neurodevelopment. There were no differences in odds for either domain by maternal ART initiation timing.

**Conclusions:**

Delayed neurodevelopment was detected in both gross and fine motor functions in this cohort of HEU children, with strong maternal predictors that may be explored as potentially modifiable factors associated with neurodevelopment at one to two years of age.

## Introduction

Although antiretroviral therapy (ART) has been highly successful in preventing mother-to-child human immunodeficiency virus (HIV) transmission, there are more than 1 million children born annually to HIV-infected mothers with growing concerns regarding the health and neurodevelopment of HIV-exposed uninfected (HEU) children [1–4]. Poor early childhood neurodevelopment is linked to educational under-achievement and lifetime progression overall, contributing to high levels of inequality and poverty in low- and middle-income countries (LMICs) [5]. Regardless of HIV/ART exposure, LMICs are home to a substantial number of children who fail to reach their full development potential due to poverty and unstimulating environments [6]. This suggests that interventions targeted at improving maternal factors, including those related to home environment may make a difference in neurodevelopment outcomes of these already vulnerable children.

The first 1000 days from conception to two years of age is a critical time of substantial growth including 80% of brain development [7, 8]. This period presents a window to establish strong foundations that may improve the child’s early and late neurodevelopment outcomes, thereby positively setting the stage for success across multiple outcomes in later life. In particular, interventions that target child neurodevelopment are most effective for children when they are still young [5]. In high income countries, appropriate neurodevelopmental learning opportunities have shown significant benefits including improved cognitive function, school achievement and increased earnings [9, 10]. In sub-Saharan Africa (SSA), behavioural programs promoting child-parent/caregiver interaction and combined infant/young child feeding, improved water, sanitation and hygiene are recommended for minimizing the risk of poor child development, especially in children affected by HIV [11, 12].

Three aspects of first 1000 days crucially influencing development are nutrition and health, love and attention, play and stimulation [13]. Despite the growing number of HEU children, there are few insights on their neurodevelopment assessment using parent-centric tools which promote interaction between mother/caregiver and child, and may encourage parents to provide stimulating environments through play and learning activities to influence neurodevelopment in their kids. HEUs from SSA most commonly experience delay in motor and language scores [1, 14–17], with exposure to efavirenz (EFV) regimen associated with worse delay in motor development compared to non-EFV regimen [15]. Further, earlier rather than later maternal ART initiation has been implicated in worse outcomes on HEU development [15], although this has not been confirmed in other studies which also suggested that maternal ART exposure may become less important in predicting child’s development with increasing child age [12, 18]. Therefore, further investigation of the role of ART exposure during pregnancy and timing of initiation on HEU development is needed. In addition, there is a need for identification of maternal and child factors that may be modified to improve neurodevelopment at a young age, particularly those that would enable physical and mental stimulation. In a cohort of HEU children, whose mothers initiated ART pre- or during pregnancy, we examined their neurodevelopment at 12–24 months of age using Ages and Stages Questionnaire (ASQ), a neurodevelopmental assessment tool designed to be completed by parents/caregivers [19], and associations with maternal and child factors.

## Methods

The study was approved by the Faculty of Health Sciences Human Research Ethics Committee of the University of Cape Town and Institutional Review Board of the University of Southampton. Written informed consent for data collection was obtained from all participants at enrolment, including consent for follow-up of children soon after delivery. Cohort details have been described elsewhere [20]. Briefly, we enrolled 552 HIV-infected, pregnant women (≥18 years) attending their first antenatal care (ANC) visit at ≤24 weeks gestational age (GA) at Gugulethu Community Health Centre (CHC). Enrolment took place between April 2015 and October 2016 and participants were prospectively followed via face-to-face study visits at the UCT-research facility located at Gugulethu CHC through May 2018. There were three antenatal (≤24, 28–32 and 34–36 weeks GA) and four postnatal (<7 days, 10 weeks, 6 and 12–24 months) study visits. The 12–24 months visit took place between March 2017 and May 2018, and ASQ assessments were also conducted at the UCT-research facility located at Gugulethu CHC. Gugulethu is a semi-urban area with a population predominantly made up of 98.8% black African ethnic group with low socioeconomic status (SES) [21, 22]. Women initiated ART pre- (n = 261) or during (n = 291) pregnancy; all were followed to 12–24 months postpartum.

**Fig 1:**
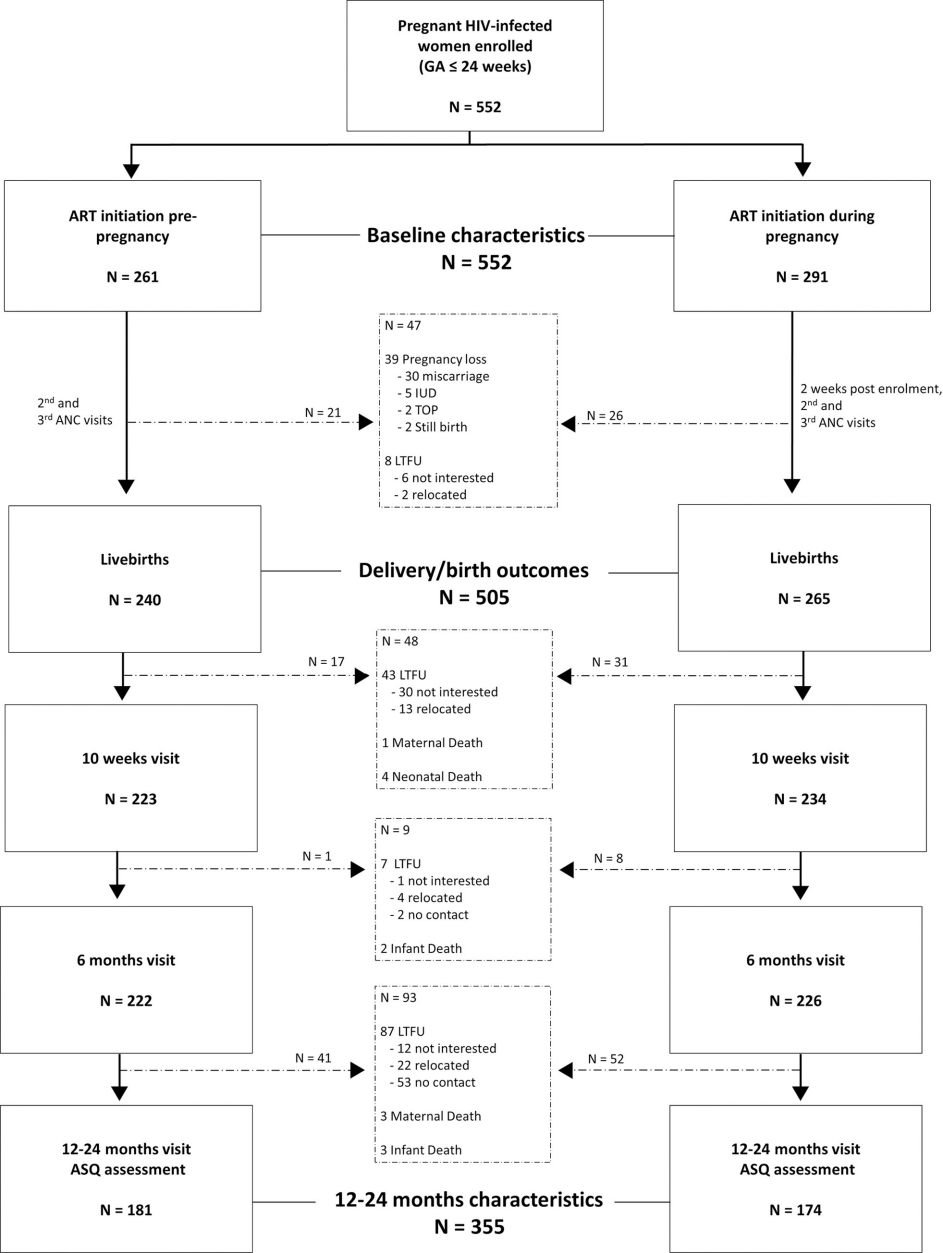
Flow diagram showing participant enrolment and retention at different study visits by maternal ART initiation status. GA—gestational age, ART—antiretroviral therapy, IUD—intrauterine death, TOP—termination of pregnancy, ANC—antenatal care, LTFU—loss to follow up, ASQ—Ages & Stages Questionnaire.

Maternal socio-demographic and clinical data were collected via interviewer-administered questionnaires. SES was a composite score based on education level, employment status, type of housing, and presence of a toilet, running water, electricity, fridge, telephone and television in the house [23]; participants were of generally low SES and we categorised into tertiles corresponding to lowest, middle and highest SES group. Substance use combined use of alcohol, cigarette and drugs 30 days prior enrolment. Neonatal data including weight, length, head circumference and gender were obtained from medical records. GA at first ANC visit was measured by ultrasonography (USS) operated by an experienced sonographer. Maternal weight and height measurements were taken at first ANC visit; weight measured at first ANC visit was corrected [24] to estimate pre-pregnancy body mass index (BMI, kg/m^2^), which was categorised as underweight (<18.5), normal (18.5–24.9), overweight (25–29.9) or obese (≥30). Using a standardised protocol, child anthropometry (weight, length, head circumference, mid-upper arm circumference [MUAC]) was measured by a trained study nurse at all postpartum study visits. Self-reported maternal ART adherence at 12–24 months was defined as not missing taking ART medication in the past 30 days. Of the 552 women enrolled, 39 had pregnancy losses and 8 were loss-to-follow-up (LTFU) resulting in inclusion of 505 women with live births and their 355 children assessed for neurodevelopment outcomes at 12–24 months using age-specific ASQ (Fig 1). Missing categories were included in frequency tables, and in the reference category in regressions as appropriate.

### Outcome assessment

Ages and Stages Questionnaire is a global screening scale previously used in South Africa [25–27], including validation in preterm and LBW children in other settings [28, 29]. The age-specific questionnaires were translated into the local language isiXhosa by our experienced translator (English-isiXhosa); this was validated by having a second independent translator (isiXhosa-English) translate the isiXhosa version back to English and the back translated English version matched with the original ASQs. Age-specific ASQ versions used in this study ranged from 11–26 months. To ensure reliability of the instrument, all assessments were done by a single trained fieldworker. With confirmation from the mother/caregiver, the assessor ensured that children were not sick, and were well-fed and rested prior to conducting the assessment. To facilitate accurate assessment, as much as possible, the mother/caregiver provided instruction to the child to ascertain their ability to perform the task. This was deemed sufficient as no task required the child to interact with peers. All communication between the fieldworker, participant and child was in local language, isiXhosa.

The ASQ screens five neurodevelopmental areas–gross motor, fine motor, communication, problem-solving and personal-social domains. Gross motor assesses use of large muscles including arms and legs while fine motor assesses coordination and movement of hands and fingers. Communication scale assesses language including what a child is able to say and what they can understand from the instructions they are given. Problem-solving domain assesses ability to solve problems through playing games and using toys; personal-social domain assesses self-help skills and interaction with parent/caregiver.

Each domain had six questions, each with a choice of three responses–‘not yet’, ‘sometimes’ and ‘yes’ corresponding to scores of 0, 5 and 10, respectively. The summary score for each of the five domains provided a total score of 0–60. Scoring was divided into three neurodevelopment categories as defined in the age-specific ASQ manual–below cutoff (delay), monitoring zone (intermediate), and above cutoff (no delay). Given the small numbers available, related domains were combined in the regression models: gross and fine motor (gross-fine motor), and communication with problem solving and personal-social (communication-problem-solving-personal-social). Detailed associations for each neurodevelopment domain are presented in supplementary material. Finally, the scores below cutoff (delay) and on monitoring zone (intermediate) were collapsed into one category of delayed neurodevelopment.

### Statistical analysis

Data were analysed using STATA version 15.0 (Stata Corporation, College Station, TX, USA). Maternal and child baseline and 12–24 months characteristics were stratified by maternal ART initiation status, differences between groups were compared using Chi-Squared test for categorical variables and Wilcoxon rank-sum test for continuous variables. To assess factors associated with LTFU between enrolment in early pregnancy and 24 months postpartum, we used univariate and multivariable logistic regression models. Associations between maternal, child characteristics and neurodevelopment outcomes, were also assessed in logistic regression—with ‘no delay’ in neurodevelopment as reference category. Results are presented as unadjusted (OR) and adjusted odds ratios (aOR) with related 95% confidence intervals (CI). Model for maternal factors was adjusted for age, BMI, SES and ART initiation status; the model for child factors was adjusted for gender, size for GA, delivery GA, age and weight-for-age at assessment. Variables included in adjusted models were those significantly (p<0.05) associated with neurodevelopment in unadjusted models, or on the basis of existing literature, theoretical and conceptual reasoning.

## Results

The median maternal age was 30 years (IQR, 26–34), 23% were nulliparous, 52% initiated ART during pregnancy, and the median CD4 count was 436 cells/μl (IQR, 305–604) (Table 1). Baseline characteristics did not differ between the 505 women with live singleton births and the 355 women whose children were assessed for neurodevelopment using ASQ at 12–24 months postpartum. Overall (n = 505), 16% children had low birth weight (LBW), 15% were small size for gestational age (SGA), and 14% were preterm (6% spontaneous preterm delivery—sPTD, 8% medically-indicated preterm delivery—MI PTD) (Table 1). Six percent of children were never breastfed; 50% breastfed for <6 months. For women whose children were assessed at 12–24 months, those who initiated ART during pregnancy were more likely younger, nulliparous and with lower CD4 counts than those who initiated ART pre-pregnancy (Table 1). The children assessed for neurodevelopment had a mean age of 14 months (SD, ±3) and median age of 12 months (IQR, 12–15). Children of mothers initiating ART during pregnancy were more likely breastfed than those of mothers initiating ART pre-pregnancy. Between birth and age 12–24 months, 13% of children had at least one hospital admission, and 52% missed at least one vaccination dose. Child characteristics at birth did not differ for the 505 liveborn and 355 children assessed for neurodevelopment at 12–24 months of age.

**Table 1:** Characteristics for women with live singleton births and their children (n = 505) and for women who had their children assessed for neurodevelopment with ASQ at 12–24 months and their children (n = 355) stratified by maternal ART initiation status.

			ART initiation status		
Characteristics	Total for livebirths	Total for children assessed at 12-24m	Pre-Pregnancy	During Pregnancy	p- value
	N (%) (n = 505)	N (%) (n = 355)	N (%) (n = 181)	N (%) (n = 174)	
**Maternal**					
***At baseline***					
Age (years)					**<0.001**
<24	88 (17)	44 (12)	18 (10)	26 (15)	
25–29	144 (29)	100 (28)	36 (20)	64 (37)	
30–34	163 (32)	123 (35)	61 (34)	62 (36)	
≥35	110 (22)	88 (25)	66 (36)	22 (13)	
Median (IQR)	30 (26–34)	31 (27–34)	33 (29–36)	29 (26–32)	
BMI (kg/m^2^)					0.397
Underweight (<18.5)	10 (2)	6 (2)	4 (2)	2 (1)	
Normal (18.5–24.9)	129 (26)	88 (25)	38 (21)	50 (29)	
Overweight (25–29.9)	109 (22)	90 (25)	51 (28)	39 (22)	
Obese (≥30)	227 (45)	156 (44)	81 (45)	75 (43)	
Missing	30 (6)	15 (4)	7 (4)	8 (5)	
Median (IQR)	30 (24–34)	29 (25–34)	29 (25–34)	29 (24–35)	
Relationship Status					0.095
*M-Living together/cohabiting	249 (49)	176 (50)	93 (51)	83 (48)	
*M-Not living together/not cohabiting	240 (48)	167 (47)	86 (48)	81 (47)	
Not in a relationship	12 (3)	9 (3)	2 (1)	7 (4)	
Missing	4 (1)	3 (1)	0	3 (2)	
SES					0.071
Lower	160 (32)	107 (30)	63 (35)	44 (25)	
Middle	150 (30)	108 (30)	47 (26)	61 (35)	
Higher	186 (37)	135 (38)	70 (39)	65 (37)	
Missing	9 (2)	5 (1)	1 (1)	4 (2)	
*Substance use					0.222
Yes	108 (21)	79 (22)	35 (19)	44 (25)	
No	391 (77)	271 (76)	142 (78)	129 (74)	
Missing	6 (1)	5 (1)	4 (2)	1 (1)	
Parity					**0.033**
Nulliparous	117 (23)	73 (21)	29 (16)	44 (25)	
Multiparous	380 (75)	279 (79)	150 (83)	129 (74)	
Missing	8 (2)	3 (1)	2 (1)	1 (1)	
Median (IQR)	1 (1–2)	1 (1–2)	1 (1–2)	1 (0–2)	
ART initiation status					
During pregnancy	265 (52)	174 (49)	-----------	-----------	
Pre-pregnancy	240 (48)	181 (51)			
CD4 cell count (cells/μl)					**<0.001**
Missing	99 (20)	69 (19)	28 (15)	41 (24)	
Median (IQR)	436 (305–604)	452 (313–609)	534 (385–663)	371 (245–502)	
***At child’s assessment***					
ART Adherence					0.821
Adherent	338 (67)	319 (90)	162 (89)	157 (90)	
Default	40 (8)	36 (10)	19 (11)	17 (10)	
Missing	127 (25)	0	0	0	
**Child**					
***At birth***					
Gender					0.167
Male	270 (53)	199 (56)	95 (52)	104 (60)	
Female	228 (45)	156 (44)	86 (48)	70 (40)	
Missing	7 (1)	0	0	0	
Birthweight (g)					0.494
Low (<2500)	82 (16)	57 (16)	31 (17)	26 (15)	
Normal (2500–4000)	395 (78)	283 (80)	145 (80)	138 (79)	
High (>4000)	20 (4)	13 (4)	4 (2)	9 (5)	
Missing	8 (2)	2 (1)	1 (1)	1 (1)	
Median (IQR)	3120 (2710–3430)	3100 (2735–3420)	3100 (2695–3400)	3100 (2750–3450)	
Size for GA (percentile)					0.425
Small (<10^th^)	78 (15)	56 (16)	33 (18)	23 (13)	
Appropriate (10-90^th^)	374 (74)	270 (76)	135 (75)	135 (78)	
Large (>90^th^)	43 (9)	28 (8)	13 (7)	15 (9)	
Missing	10 (2)	2 (1)	0	1 (1)	
Gestation at delivery (weeks)					0.465
Term delivery (≥37)	368 (73)	272 (77)	142 (78)	130 (75)	
Spontaneous preterm (<37)	32 (6)	22 (6)	13 (7)	9 (5)	
Medically-indicated preterm (<37)	40 (8)	29 (8)	13 (7)	16 (9)	
Missing	65 (13)	32 (9)	13 (7)	19 (11)	
Head circumference (cm)					0.402
Missing	75 (15)	38 (11)	17 (9)	21 (12)	
Median (IQR)	34 (33–35)	34 (33–35)	34 (33–35)	34 (33–35)	
Length (cm)					0.653
Missing	84 (17)	43 (12)	20 (11)	23 (13)	
Median (IQR)	49 (47–52)	49 (47–52)	49 (47–51)	49 (47–52)	
***Between birth and assessment***					
Breastfeeding duration					**0.035**
Never	32 (6)	22 (6)	16 (9)	6 (3)	
Ever	334 (66)	319 (90)	158 (87)	161 (93)	
<6 months	200 (40)	178 (50)	96 (53)	82 (47)	0.531
≥6 months	166 (33)	163 (46)	78 (43)	85 (49)	
Missing	139 (28)	14 (4)	7 (4)	7 (4)	
Median (IQR)	4 (1–12)	5 (1–12)	4 (1–12)	6 (1–12)	
Hospital admission					0.652
Yes	64 (13)	60 (17)	29 (16)	31 (18)	
No	395 (78)	295 (83)	152 (84)	143 (82)	
Missing	46 (9)	0	0	0	
Missed vaccinations					0.607
Yes	263 (52)	138 (39)	68 (38)	70 (40)	
No	242 (48)	217 (61)	113 (62)	104 (60)	
***At assessment***					
Age (months)					0.461
Median (IQR)	-----------	12 (12–15)	12 (12–14)	12 (12–16)	
Weight (kg)					0.979
Median (IQR)	-----------	10.2 (9.4–11.4)	10.3 (9.4–11.3)	10.2 (9.3–11.5)	
Height (cm)					0.502
Missing	-----------	2 (1)	1 (1)	1 (1)	
Median (IQR)	-----------	76 (73–78)	76 (73–78)	76 (73–79)	
MUAC (cm)					0.54
Missing	-----------	1 (1)	1 (1)	0	
Median (IQR)	-----------	16 (15–17)	16 (15–17)	16 (15–17)	
Weight-for-age (g)					0.131
Median (IQR)	-----------	0.47 (-0.31, 1.37)	0.59 (-0.22, 1.50)	0.41 (-0.36, 1.23)	
Height-for-age (cm)					0.226
Missing	-----------	2 (1)	1 (1)	1 (1)	
Median (IQR)	-----------	-0.6 (-1.36, 0.19)	-0.43 (-1.38, 0.38)	-0.73 (-1.35, 0.06)	
Weight-for-height					0.234
Missing	-----------	3 (1)	1 (1)	2 (1)	
Median (IQR)	-----------	1.02 (0.18–1.91)	1.10 (0.24–1.99)	0.97 (0.16–1.81)	
Head circumference (cm)					0.186
Missing	-----------	1 (1)	1 (0)	0	
Median (IQR)	-----------	47 (46–48)	47 (46–48)	47 (46–48)	
ASQ version used (months)					0.126
11–13	-----------	246 (69)	131 (72)	115 (66)	
15–17	-----------	38 (11)	22 (12)	16 (9)	
17–19	-----------	42 (12)	16 (9)	26 (15)	
20–23	-----------	18 (5)	8 (4)	10 (6)	
24–26	-----------	4 (1)	0	4 (2)	
Missing	-----------	7 (2)	4 (2)	3 (2)	

BMI—body mass index, SES—socioeconomic status, ART—antiretroviral therapy, GA—gestational age, MUAC—mid-upper arm circumference, ASQ—Ages & Stages Questionnaire.

*M-Living together/cohabiting—married and living together/ not married but cohabiting, *M-Not living together/not cohabiting—married but not living together, not married and not cohabiting, *Substance use—combination of alcohol, cigarette and drug use 30 days prior enrolment.

The majority (17%) of LTFU occurred between 6 and 24 months visits. To promote retention, participants were contacted twice every 1–2 months through telephone and home visits were conducted for those unreachable over the phone. In adjusted analyses, odds of LTFU were lower for women 30–34 years old (aOR 0.46, 95% CI 0.23–0.92), overweight women (aOR 0.40, 95% CI 0.18–0.88) and those who initiated ART pre-pregnancy (aOR 0.29, 95% CI 0.17–0.51) (S2 Table). Adjusted factors non-significantly associated with increased LTFU odds included underweight BMI (aOR 3.05, 95% CI 0.81–11.44) and higher maternal SES (aOR 1.48, 95% CI 0.81–2.73); and substance use 30 days prior enrolment (OR 1.16, 95% CI 0.67–2.02) in unadjusted model.

**Fig 2:**
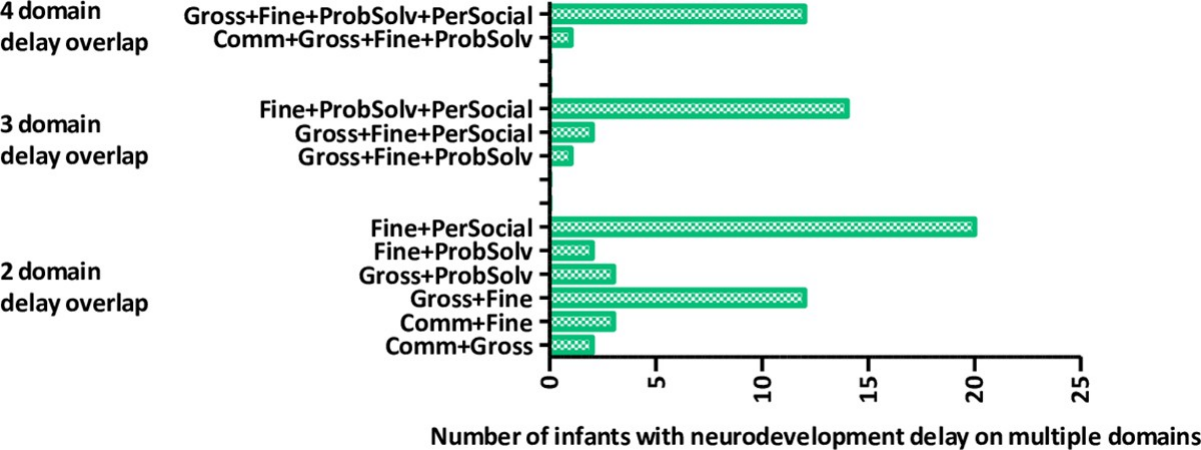
Distribution of delayed neurodevelopment on two, three and four overlapping domains. Neurodevelopment delay overlap on two domains: Fine+PerSocial: fine motor & personal social; Fine+ProbSolv: fine motor & problem solving; Gross+ProbSolv: gross motor & problem solving; Gross+Fine: gross & fine motor; Comm+Fine: communication & fine motor; Comm+Gross: communication & gross motor. Neurodevelopment delay overlap on three domains: Fine+ProbSolv+PerSocial: fine motor & problem solving & personal social; Gross+Fine+PerSocial: gross motor & fine motor & personal social; Gross+Fine+ProbSolv: gross motor & fine motor & problem solving. Neurodevelopment delay overlap on four domains: Gross+Fine+ProbSolv+PerSocial: gross motor & fine motor & problem solving & personal social; Comm+Gross+Fine+ProbSolv: communication & gross motor & fine motor & problem solving.

Overall, 9% of children had delayed neurodevelopment on gross motor, 5% on fine motor, 3% on communication and problem-solving and 4% on personal-social domains (Table 2); with no substantive differences by maternal ART initiation status except for gross motor. Children of women initiating ART during pregnancy appeared less likely to have delayed neurodevelopment (combined intermediate and delay categories) than those of women initiating pre-pregnancy (13% vs 17%). Notably, delayed neurodevelopment overlapped across different domains. 42 children had delay in both fine motor and personal-social domains, 17 in fine motor, problem solving and personal-social domains and 13 in gross motor, fine motor, problem solving and personal-social domains (Fig 2).

**Table 2:** Frequencies of individual ASQ neurodevelopment domains stratified by maternal ART initiation status (n = 355).

		ART initiation status		
Neurodevelopment Sub-scale	Total	Pre-pregnancy	During pregnancy	p- value
	N (%) (n = 355)	N (%) (n = 181)	N (%) (n = 174)	
Gross motor				0.052
No delay	303 (85)	151 (83)	152 (87)	
Intermediate	21 (6)	16 (9)	5 (3)	
Delay	31 (9)	14 (8)	17 (10)	
Median (IQR)	60 (50–60)	60 (50–60)	60 (50–60)	
Fine motor				0.943
No delay	282 (79)	144 (80)	138 (79)	
Intermediate	54 (15)	28 (15)	26 (15)	
Delay	19 (5)	9 (5)	10 (6)	
Median (IQR)	50 (45–60)	50 (45–60)	50 (45–60)	
Communication				0.777
No delay	331 (93)	169 (93)	162 (93)	
Intermediate	11 (3)	5 (3)	6 (3)	
Delay	12 (3)	6 (3)	6 (3)	
Median (IQR)	55 (45–60)	55 (45–60)	53 (50–60)	
Problem-solving				0.9
No delay	335 (94)	171 (94)	164 (94)	
Intermediate	9 (3)	5 (3)	4 (2)	
Delay	11 (3)	5 (3)	6 (3)	
Median (IQR)	60 (50–60)	60 (50–60)	60 (50–60)	
Personal-social				0.126
No delay	319 (90)	156 (86)	163 (94)	
Intermediate	18 (5)	13 (7)	5 (3)	
Delay	14 (4)	9 (5)	5 (3)	
Median (IQR)	50 (45–60)	50 (45–60)	50 (45–60)	

### Maternal factors and neurodevelopment at 12–24 months

Table 3 shows the association between maternal, child factors and delayed neurodevelopment on combined ASQ domains (results on individual domains available as S6–S9 Tables). Adjusting for age, BMI, SES and ART initiation status, maternal factors associated with a (non-significant) trend towards increased odds of delayed gross-fine motor neurodevelopment included underweight BMI (aOR 2.64, 95% CI 0.51–13.71), lower SES (aOR 1.08, 95% CI 0.60–1.93) and ART initiation pre-pregnancy (aOR 1.20, 95% CI 0.72–1.97). Higher maternal SES was the only factor statistically significantly associated with reduced risk (aOR 0.42, 95% CI 0.24–0.76) of delayed gross-fine motor neurodevelopment.

**Table 3:** Associations between maternal, child factors and delayed neurodevelopment on combined ASQ domains, adjusted maternal and child factors had two separate models (n = 355).

		ASQ Neurodevelopment Domains (Reference category–No delay)							
		Unadjusted OR’s				Adjusted OR’s			
	Total	Gross + Fine motor		Comm + ProbSolv + PerSocial		Gross + Fine motor		Comm + ProbSolv + PerSocial	
Characteristics	N (%)	OR (95% CI)	p-value	OR (95% CI)	p-value	aOR (95% CI)	p-value	aOR (95% CI)	p-value
**Maternal**									
***At baseline***									
Age (years)									
<24	44 (12)	1.00 (ref)		1.00 (ref)		1.00 (ref)		1.00 (ref)	
25–29	100 (28)	1.14 (0.52–2.52)	0.741	0.93 (0.35–2.48)	0.889	1.22 (0.55–2.71)	0.623	0.99 (0.37–2.66)	0.98
30–34	123 (35)	0.93 (0.43–2.04)	0.871	0.91 (0.35–2.35)	0.839	0.92 (0.43–2.00)	0.841	0.84 (0.32–2.21)	0.727
≥35	88 (25)	1.00 (0.44–2.26)	1	0.53 (0.18–1.57)	0.251	0.93 (0.41–2.12)	0.857	0.44 (0.14–1.37)	0.157
BMI (kg/m^2^)									
Normal (18.5–24.9)	88 (25)	1.00 (ref)		1.00 (ref)		1.00 (ref)		1.00 (ref)	
Underweight (<18.5)	6 (2)	2.32 (0.44–12.18)	0.319	3.18 (0.53–19.06)	0.206	2.64 (0.51–13.71)	0.247	3.04 (0.54–17.13)	0.207
Overweight (25–29.9)	90 (25)	0.89 (0.48–1.67)	0.724	1.27 (0.58–2.81)	0.552	0.87 (0.46–1.64)	0.663	1.23 (0.56–2.71)	0.613
Obese (≥30)	156 (44)	0.77 (0.44–1.35)	0.367	0.78 (0.36–1.66)	0.515	0.75 (0.42–1.33)	0.327	0.81 (0.37–1.74)	0.586
Relationship Status									
*M-Not living together/not cohabiting	167 (47)	1.00 (ref)		1.00 (ref)					
*M-Living together/cohabiting	176 (50)	1.36 (0.85–2.19)	0.201	1.02 (0.55–1.90)	0.564				
No relationship	9 (3)	0.88 (0.18–4.04)	0.874	1.88 (0.37–9.67)	0.448				
SES									
Middle	108 (30)	1.00 (ref)		1.00 (ref)		1.00 (ref)		1.0 (ref)	
Lower	107 (30)	1.09 (0.62–1.90)	0.774	0.99 (0.46–2.12)	0.976	1.08 (0.60–1.93)	0.797	1.00 (0.45–2.25)	0.995
Higher	135 (38)	**0.44 (0.25–0.80)**	**0.007**	0.87 (0.42–1.82)	0.718	**0.42 (0.24–0.76)**	**0.004**	0.82 (0.38–1.74)	0.602
*Substance use									
No	271 (76)	1.00 (ref)		1.00 (ref)					
Yes	79 (22)	0.66 (0.36–1.20)	0.172	1.36 (0.68–2.71)	0.389				
Parity									
Nulliparous	73 (21)	1.00 (ref)		1.00 (ref)					
Multiparous	279 (79)	1.19 (0.66–2.12)	0.567	0.79 (0.39–1.61)	0.516				
ART initiation status									
During pregnancy	174 (49)	1.00 (ref)		1.00 (ref)		1.00 (ref)		1.00 (ref)	
Pre-pregnancy	181 (51)	1.12 (0.70–1.79)	0.63	1.56 (0.84–2.90)	0.163	1.20 (0.72–1.97)	0.486	1.81 (0.97–3.38)	0.062
***At child’s assessment***									
ART Adherence									
Adherent	319 (90)	1.00 (Ref)		1.00 (Ref)					
Default	36 (10)	1.78 (0.87–3.64)	0.114	0.78 (0.26–2.32)	0.657				
**Child**									
***At birth***									
Gender									
Male	199 (56)	1.00 (Ref)		1.00 (Ref)		1.00 (Ref)		1.00 (Ref)	
Female	156 (44)	0.70 (0.44–1.13)	0.148	**0.48 (0.25–0.93)**	**0.029**	0.68 (0.40–1.15)	0.148	0.58 (0.28–1.18)	0.132
Birthweight (g)									
Normal (2500–4000)	283 (80)	1.00 (Ref)		1.00 (Ref)					
Low (<2500)	57 (16)	1.27 (0.68–2.35)	0.45	1.65 (0.78–3.49)	0.186				
High (>4000)	13 (4)	1.22 (0.37–4.09)	0.745	0.58 (0.07–4.58)	0.602				
Size for GA (percentile)									
Appropriate (10-90^th^)	270 (76)	1.00 (Ref)		1.00 (Ref)		1.00 (Ref)		1.00 (Ref)	
Small (<10^th^)	56 (16)	0.82 (0.43–1.60)	0.568	1.21 (0.55–2.68)	0.636	0.78 (0.37–1.64)	0.505	1.45 (0.61–3.43)	0.402
Large (>90^th^)	28 (8)	0.67 (0.26–1.73)	0.413	0.49 (0.11–2.14)	0.34	0.71 (0.26–1.96)	0.506	0.59 (0.14–2.52)	0.478
Gestation at delivery (weeks)									
Term delivery (≥37)	272 (77)	1.00 (Ref)		1.00 (Ref)		1.00 (Ref)		1.00 (Ref)	
Spontaneous preterm (<37)	22 (6)	1.65 (0.66–4.10)	0.282	1.67 (0.53–5.24)	0.382	1.49 (0.61–3.65)	0.387	1.58 (0.49–5.09)	0.444
Medically-indicated preterm (<37)	29 (8)	1.10 (0.47–2.60)	0.829	0.56 (0.13–2.45)	0.438	1.18 (0.49–2.83)	0.708	0.54 (0.13–2.35)	0.414
Head circumference (cm)	317 (89)	0.96 (0.83–1.12)	0.619	0.87 (0.74–1.02)	0.092				
Length (cm)	312 (88)	0.95 (0.90–1.01)	0.087	0.93 (0.86–1.00)	0.047				
***Between birth and assessment***									
Breastfeeding duration									
Never	22 (6)	1.00 (Ref)		1.00 (Ref)		1.00 (Ref)		1.00 (Ref)	
Ever	319 (90)	0.99 (0.46–2.14)	0.981	0.61 (0.25–1.49)	0.278	1.23 (0.49–3.09)	0.652	1.07 (0.35–3.26)	0.91
<6 months	178 (50)	1.00 (Ref)		1.00 (Ref)					
≥6 months	163 (46)	0.72 (0.45–1.17)	0.192	**0.50 (0.26–0.97)**	**0.04**				
Hospital admissions									
No	295 (83)	1.00 (Ref)		1.00 (Ref)					
Yes	60 (17)	1.27 (0.69–2.32)	0.441	1.80 (0.87–3.71)	0.112				
Missed vaccinations									
No	217 (61)	1.00 (Ref)		1.00 (Ref)					
Yes	138 (39)	1.26 (0.78–2.02)	0.343	1.04 (0.55–1.93)	0.914				
***At assessment***									
Age	355 (100)	0.67 (0.25–1.74)	0.407	0.87 (0.23–3.26)	0.838	0.44 (0.14–1.37)	0.156	0.90 (0.20–4.02)	0.886
Weight (kg)	355 (100)	1.00 (1.00–1.01)	0.326	1.00 (1.00–1.01)	0.523				
Height (cm)	353 (99)	0.96 (0.91–1.01)	0.129	1.00 (0.94–1.07)	0.979				
MUAC (cm)	354 (99)	0.95 (0.82–1.09)	0.454	1.07 (0.91–1.26)	0.395				
Head circumference (cm)	354 (99)	0.94 (0.81–1.07)	0.339	0.98 (0.82–1.16)	0.773				
Weight-for-age (g)	355 (100)	0.94 (0.80–1.10)	0.441	1.07 (0.88–1.31)	0.501	0.91 (0.76–1.09)	0.293	1.10 (0.90–1.34)	0.375
Height-for-age (cm)	353 (99)	0.89 (0.75–1.06)	0.203	0.96 (0.81–1.14)	0.67				
Weight-for-height	352 (99)	1.00 (0.87–1.15)	0.98	1.09 (0.93–1.27)	0.273				

BMI—body mass index, SES—socioeconomic status, ART—antiretroviral therapy, GA—gestational age, MUAC—mid-upper arm circumference, ASQ—Ages & Stages Questionnaire, OR—odds ratio.

*M-Living together/Cohabiting—married and living together/ not married but cohabiting, *M-Not living together/not cohabiting—married but not living together, not married and not cohabiting, *Substance use—combination of alcohol, cigarette and drug use 30 days prior enrolment. Gross + Fine motor: combined gross motor & fine motor domains; Comm + ProbSolv + PerSocial: combined communication & problem solving & personal social domains. Maternal model adjusted for age, BMI, SES and ART initiation status. Chid model adjusted for gender, size for GA, delivery GA, breastfeeding duration and weight-for-age at assessment. Missing data for n = 355, n (%): BMI n = 15 (4.2), Relationship status n = 3 (0.9), SES and Substance use n = 5 (1.4), Parity and ART adherence at child’s assessment n = 3 (0.9), Birthweight, Height at assessment and Height-for-age n = 2 (0.6), Size for GA, Breastfeeding and Head circumference at assessment n = 1 (0.3), Birth head circumference n = 38 (10.7), Birth length n = 43 (12.1), Weight-for-height n = 3 (0.8), ASQ version n = 7 (2.0). Where data are missing on predictors, cases were included in the reference category in the regression. Interpretation of OR’s for categorical predictors: Predictor was associated with increased (OR>1) or decreases (OR<1) odds of having delayed (domain name) neurodevelopment compared to reference category (for that predictor). Interpretation of OR’s for continuous predictors: Unit increase in predictor was associated with increased (OR>1) or decreases (OR<1) odds of having delayed (domain name) neurodevelopment.

In adjusted models, no factors were significantly associated with communication-problem-solving-personal-social neurodevelopment, although there was a trend towards increased odds of delayed neurodevelopment on this combined domain for underweight BMI (aOR 3.04, 95% CI 0.54–17.13) and ART initiation pre-pregnancy (aOR 1.81, 95% CI 0.97–3.38). Factors showing a trend towards decreased odds of delayed communication-problem-solving-personal-social neurodevelopment included older maternal age (aOR 0.44, 95% CI 0.14–1.37), obese BMI (aOR 0.81, 95% CI 0.37–1.74) and higher maternal SES (aOR 0.82, 95% CI 0.38–1.74) in adjusted model. In model adjusted for both maternal and child factors, older maternal age (aOR 0.22, 95% CI 0.05–0.91) significantly reduced odds of communication-problem-solving-personal-social neurodevelopment delay, while underweight BMI (aOR 6.72, 95% CI 1.05–43.00) increased the odds (S3 Table).

### Child factors and neurodevelopment at 12–24 months

In a model adjusted for child gender, size for GA, delivery GA, age and weight-for-age at assessment (Table 3), there was a (non-significant) trend towards increased odds of delayed gross-fine motor neurodevelopment for sPTD (aOR 1.49, 95% CI 0.61–3.65) and MI PTD (aOR 1.18, 95% CI 0.49–2.83). Factors non-significantly associated with decreased odds of delayed gross-fine motor neurodevelopment included female gender (aOR 0.68, 95% CI 0.40–1.15) and large size-for-gestational age (LGA) (aOR 0.71, 95% CI 0.26–1.96); breastfeeding for ≥6 months (OR 0.72, 95% CI 0.45–1.17) was non-significant in unadjusted model.

In adjusted models, factors with non-significant increased odds of delayed communication-problem-solving-personal-social neurodevelopment included small size-for-gestational age (SGA) (aOR 1.45, 95% CI 0.61–3.43) and sPTD (aOR 1.58 95% CI 0.49–5.09). Although female gender (OR 0.48, 95% CI 0.25–0.93) and breastfeeding for ≥6 months (OR 0.50, 95% CI 0.26–0.97) were associated with decreased odds of delayed communication-problem-solving-personal-social neurodevelopment in unadjusted models, significance was lost in adjusted models.

### Neurodevelopment of SGA children at 12–24 months

Of the 355 children assessed at 12–24 months, 16% were SGA, (18% for mothers initiating ART pre-pregnancy, 13% for those initiating ART during pregnancy) (Table 1). We analysed frequencies of delayed neurodevelopment on different ASQ domains (S4 Table) and associations with maternal factors in 56 SGA children (S5 Table). Of these 56 children, 11% had delayed neurodevelopment on gross motor, 9% on fine motor and personal-social, and 5% on communication and problem-solving (S4 Table). Although not statistically significant, children of mothers initiating ART pre-pregnancy had notably higher frequencies of delay in all domains than those of mothers initiating ART during pregnancy, similar to what was seen in the overall 355 cohort.

In unadjusted models there was a trend for underweight BMI (OR 2.29, 95% CI 0.12–43.11) and initiating ART pre-pregnancy (OR 1.35, 95% CI 0.38–4.78) to be associated with increased odds of delayed gross-fine motor neurodevelopment (S5 Table). Factors with non-significant decreased odds of delayed gross-fine motor neurodevelopment in SGA children included older maternal age (OR 0.67, 95% CI 0.11–3.90), obese BMI (OR 0.40, 95% CI 0.09–1.86), being married and living together/cohabiting (OR 0.44, 95% CI 0.02–8.25), higher SES (OR 0.71, 95% CI 0.17–2.98) and multiparity (OR 0.72, 95% CI 0.20–2.62). Except for maternal age, obese BMI and relationship status, associations with communication-problem-solving-personal-social domain combination were in the same direction as gross-fine motor domain and not substantially different to those observed in the overall cohort (n = 355).

## Discussion

In HEU children of mothers who initiated ART pre- or during pregnancy, delayed neurodevelopment at age one to two years was limited, and mostly on gross or fine motor functions. Children of higher SES mothers were less likely to have delayed gross-fine motor neurodevelopment. Children breastfed for ≥6 months and children of mothers ≥35 years of age were less likely, and those of underweight BMI mothers more likely, to have delayed communication-problem-solving-personal-social neurodevelopment. This data would suggest potentially modifiable factors to improve neurodevelopment of HEU children.

Various tools are used to assess child neurodevelopment, some administered by health professionals and others by parents/caregivers. ASQ is a globally-used scale, cheap and easy to administer, and increasingly popular in LMICs [19, 30]. The parent-centric nature of ASQ makes it a convenient and appropriate tool for use in LMICs, where it is needed the most [6]. Although some studies have questioned the weak correlation between ASQ and Bayley scale for children under 13 months [30, 31], ASQ provides a critical snapshot to child’s neurodevelopment, and can identify early delays, enabling timely provision of appropriate learning activities. Research has recently validated the use of this screening tool in South Africa [25, 26] and we used this tool to assess neurodevelopment outcomes in a cohort of HEU children at age one to two years.

In our cohort, gross and fine motor functions were domains where children most likely experienced neurodevelopment delay. Although we did not have a comparator group of HIV-unexposed children, the proportions for gross (9% vs 5%) and fine (5% vs 2%) motor function delays observed in HEU children in this study are higher than those reported in other studies for HIV-unexposed children in a similar setting in Cape Town [1, 16, 32]. Development of these functions can be stimulated by activities including sitting, standing, walking, eating, drawing and general playing [33]. In LMICs, neurodevelopment delays may be attributed to multiple risks factors regardless of ART exposure. We found that children of mothers with higher SES were less likely to experience neurodevelopment delay in these domains; higher SES may provide a healthy and stimulating home environment, with positive impact on child neurodevelopment [34–37]. Other African studies also report higher maternal SES to be positively associated with child gross-fine motor neurodevelopment [38, 39], which may be partly due to educated mothers being knowledgeable about the importance of providing stimulating environment for their children, and those employed able to afford physically-stimulating learning activities. Higher maternal SES may mediate child neurodevelopment through improved child nutrition [39]. Although our results for motor function delays are comparable with other cohorts of HEU children in Cape Town, the most common delay reported in these studies is the communication domain rather than motor function [1, 16, 32]. These differences may be, in part, attributed to different assessment tools used. Overall, these results suggest that improving the factors included in SES could indirectly provide a home environment that promotes healthy growth and general play, stimulating gross and fine neurodevelopment in children, including those HEU.

Three areas in the first 1000 days critical for development are nutrition and health, love and attention, play and stimulation [13]. We found that children breastfed for ≥6 months were significantly less likely to experience delay in communication-problem-solving-personal-social combination domain. Although a previous study in our setting showed that HEU children experience neurodevelopment delays despite breastfeeding, this was particularly true for preterm children [1]. In other African settings, there is evidence of beneficial effect of breastfeeding in HEU children especially during first year of life [40, 41]. We also found that children of women who were underweight were significantly more likely to have delayed communication-problem-solving-personal-social neurodevelopment. Maternal underweight BMI is a proxy for undernutrition, which may be an indication of the child’s household environment; maternal undernutrition is negatively associated with children brain development [42]. In contrast, we found that children born from older women were significantly less likely to have delayed communication-problem-solving-personal-social neurodevelopment. Older women tend to be multiparous and we speculate that they may be more capable of providing nurturing care and interaction with their children, which could stimulate their verbal and social skills. In another study in a high HIV prevalence area, Bland et al. found that home stimulation improves executive function at 11 years of age [43], and it is possible that interventions targeting modifiable factors such as maternal SES, BMI and breastfeeding may improve nutrition, interaction and play with children at one to two years of age with longer-term impact.

Despite the undisputed success of universal ART in reducing mother to child HIV transmission, concerns have been expressed regarding ART exposure on growth and neurodevelopment of HEU children [15, 44]. We observed a strong, non-significant trend for the association between ART initiation pre-pregnancy and delayed neurodevelopment in all domain combinations. This result was unexpected given that studies elsewhere have reported similar neurodevelopment progression in HEU children as seen in their unexposed counterparts [16, 18, 45]. However, cases of poor neurodevelopment in HEU children have also been reported, with ART exposure implicated as the likely contributing factor [15, 46–48]. These inconsistent findings may be attributed to the heterogeneity of regimens and ART treatment guidelines used in different studies, and over time. In our study, nearly all women were on an ART regimen of two NRTIs and EFV. Reassuringly, there is some indication that maternal ART becomes less important in predicting children’s development with increasing child age [17, 47]. Women initiating ART pre-pregnancy were significantly less likely to be LFTU, which may have biased our results, and further research remains needed.

HIV/ART has been shown to contribute to high risk of SGA and preterm children [49], we observed that women initiating ART pre-pregnancy had higher proportions of SGA children than those initiating ART during pregnancy. SGA children are likely to have delayed organ development including the brain [50–52], which may have contributed to our observation of delayed communication-problem-solving-personal-social neurodevelopment, although statistical significance was not reached due to limited sample size. Some studies report absence of adverse neurodevelopmental outcomes in preterm children [53, 54]; we show a non-significant trend for children of mothers with sPTD, but not MI PTD, to be more likely to have neurodevelopment delays in this same domain combination, which is in contrast with findings elsewhere showing higher risk of neurodevelopment delay in MI PTD than sPTD children [55]. However, that cohort had a noticeable imbalance of mode of delivery of preterm children (97.2% sPTD, 2.8% MI PTD) which could explain their findings [55]. Although the distinct risks factors mediating the two types of PTD are well established, the mechanisms underlying different risks profiles for neurodevelopment outcomes at one to two years remain unclear.

Our results support the recommendation of behavioural parent/caregiver training programs for families affected by HIV aimed at stimulating early childhood development by promoting positive experiences and happy memories which may have long-lasting effects on emotional, social and behavioural domains of the brain [12, 56]. However, the findings reported should be interpreted cautiously due to limited statistical power for both overall and subset cohort of SGA children. We were unable to control for some important confounders such as mother’s mental health status, which could have been associated with the child’s neurodevelopment, and this may have resulted in overestimation of neurodevelopment delay in the regression models. In contrast, it is possible that the neurodevelopment delays reported are less than rates in the general population as vulnerable young mothers and adolescents <18 years of age, whose children could possibly face increased neurodevelopmental delay, were not included in the main cohort [20] and this study. Sample size at 12–24 months was limited by LTFU which may have contributed to non-achievement of statistical significance. However, the trends observed highlight modifiable factors that future studies should consider investigating as neurodevelopment delays may become even more apparent as children grow older. Due to small numbers, we collapsed intermediate and delayed neurodevelopment categories, and neurodevelopment delay reported may be overestimated as children on the intermediate range may have reduced associations. However, both groups of children would require increased stimulation/learning activities to improve their neurodevelopment.

In conclusion, a small proportion of HEU children had delayed neurodevelopment in any of the domains assessed, which was less than expected from studies in the general South African population where there are many confounding factors that affect early child development. Maternal SES, BMI and breastfeeding are modifiable factors and could improve neurodevelopment of HEU children at one to two years of age. In line with WHO guidelines, these results suggest that nurturing care and good nutrition related to breastfeeding and healthy maternal BMI, as well as stimulation provided at home by parent/caregiver related to maternal SES may have a significant contribution in improving neurodevelopment of HEU children.

## Supporting information

S1 Table Characteristics for women who were LTFU and their children (n = 145). (PDF)

S2 Table Maternal factors associated with LTFU (n = 505). (PDF)

S3 Table Associations between maternal, child factors and delayed neurodevelopment on combined ASQ domains adjusted for maternal and child factors in one model (n = 355). (PDF)

S4 Table Frequencies of individual ASQ neurodevelopment domains for SGA children stratified by maternal ART initiation status (n = 56). (PDF)

S5 Table Unadjusted associations between maternal factors and delayed neurodevelopment on combined ASQ domains for SGA children (n = 56). (PDF)

S6 Table Unadjusted associations between maternal factors and delayed neurodevelopment on individual ASQ domains for SGA children (n = 56). (PDF)

S7 Table Unadjusted associations between maternal, child factors and delayed neurodevelopment on individual ASQ domains (n = 355). (PDF)

S8 Table Associations between maternal, child factors and delayed neurodevelopment on individual ASQ domains adjusted for maternal and child factors on two separate models (n = 355). (PDF)

S9 Table Associations between maternal, child factors and delayed neurodevelopment on individual ASQ domains adjusted for maternal and child factors in one model (n = 355). (PDF)
