# Supplementary material for: Neurodevelopment of HIV-exposed uninfected children in Cape Town, South Africa
Source: PLoS One. 2020 Nov 18;15(11):e0242244. doi: 10.1371/journal.pone.0242244 (PMC7673492; doi:10.1371/journal.pone.0242244)
Supplement: S1 Table — (PDF) [file pone.0242244.s001.pdf]

S1 Table. Characteristics for women who were LTFU and their children (n = 145)

Characteristics	Total N (%) (n = 145)	ART initiation status<br>Pre-Pregnancy N (%) (n = 55)	 During Pregnancy N (%) (n = 90)	p- value
**Maternal**				
At baseline				
Age (years)				**<0.001**
<24	40 (28)	6 (11)	34 (38)	
25-29	43 (30)	15 (27)	28 (31)	
30-34	38 (26)	16 (29)	22 (24)	
≥35	24 (17)	18 (33)	6 (7)	
Median (IQR)	28 (24-32)	31 (28-35)	26 (22-31)	
BMI (kg/m^2)				0.978
Underweight (<18.5)	4 (3)	1 (2)	3 (3)	
Normal (18.5-24.9)	38 (26)	14 (25)	24 (27)	
Overweight (25-29.9)	20 (14)	8 (15)	12 (13)	
Obese (≥30)	66 (46)	26 (47)	40 (44)	
Missing	17 (12)	6 (11)	11 (12)	
Median (IQR)	30 (24-34)	30 (24-33)	30 (23-34)	
Relationship Status				0.530
*M-Living together/cohabiting	68 (47)	29 (53)	39 (43)	
*M-Not living together/not cohabiting	69 (48)	24 (44)	45 (50)	
Not in a relationship	2 (1)	0	2 (2)	
Missing	6 (4)	2 (4)	4 (4)	
SES				0.398
Lower	46 (32)	16 (29)	30 (33)	
Middle	41 (28)	17 (31)	24 (27)	
Higher	50 (34)	21 (38)	29 (32)	
Missing	8 (6)	1 (2)	7 (8)	
*Substance use				0.164
Yes	30 (21)	8 (15)	22 (24)	
No	109 (75)	46 (84)	63 (70)	
Missing	6 (4)	1 (2)	5 (6)	
Parity				0.782
Nulliparous	42 (29)	15 (27)	27 (30)	
Multiparous	93 (64)	37 (67)	56 (62)	
Missing	10 (7)	3 (5)	7 (8)	
Median (IQR)	1 (0-2)	1 (0-2)	1 (0-1)	
ART initiation status				
During pregnancy	90 (62)	-----	-----	
Pre-pregnancy	55 (38)			
CD4 cell count (cells/µl)				0.071
Missing	30 (21)	12 (8)	18 (12)	
Median (IQR)	394 (300-601)	489 (344-626)	369 (278-594)	
**Child**				
At birth				0.087
Gender	66 (46)	22 (40)	44 (49)	
Male	67 (46)	31 (56)	36 (40)	
Female	12 (8)	2 (4)	10 (11)	
Missing				
Birthweight (g)				0.195
Low (<2500)	20 (14)	9 (16)	11 (12)	
Normal (2500-4000)	106 (73)	43 (78)	63 (70)	
High (>4000)	7 (5)	1 (2)	6 (7)	

Missing	12 (8)	2 (4)	10 (11)	
Median (IQR)	3160 (2760-3500)	3130 (2680-3380)	3198 (2800-3580)	
Size for GA (percentile)				0.193
Small (<10th)	20 (14)	9 (16)	11 (12)	
Appropriate (10-90th)	97 (67)	40 (73)	57 (63)	
Large (>90th)	14 (10)	4 (7)	10 (11)	
Missing	14 (10)	2 (4)	12 (13)	
Gestation at delivery (weeks)				0.164
Term delivery (≥37)	93 (64)	38 (69)	55 (61)	
Spontaneous preterm (<37)	9 (6)	3 (5)	6 (7)	
Medically-indicated preterm (<37)	10 (7)	6 (11)	4 (4)	
Missing	33 (23)	8 (15)	28 (28)	
Head circumference (cm)				0.349
Missing	38 (26)	11 (8)	27 (19)	
Median (IQR)	34 (33-36)	35 (33-36)	34 (33-36)	
Length (cm)				0.217
Missing	42 (29)	11 (8)	31 (21)	
Median (IQR)	50 (47-52)	49 (47-51)	50 (47-52)	
Between birth and assessment				0.153
Breastfeeding duration	7 (5)	3 (5)	4 (4)	
Never	17 (12)	10 (18)	7 (8)	
Ever	21 (14)	12 (22)	9 (10)	0.146
<6 months	3 (2)	1 (2)	2 (2)	
≥6 months	121 (83)	42 (76)	79 (88)	
Missing	1 (0-4)	1 (0-2)	0 (0-5)	
Median (IQR)				
Hospital admission				0.294
Yes	4 (3)	2 (4)	2 (2)	
No	93 (64)	39 (71)	54 (60)	
Missing	48 (33)	14 (25)	34 (38)	
Missed vaccinations				0.286
Yes	122 (84)	44 (80)	78 (87)	
No	23 (16)	11 (20)	12 (13)	

BMI - body mass index, SES - socioeconomic status, ART - antiretroviral therapy, GA - gestational age, MUAC - mid-upper arm circumference, ASQ - Ages & Stages Questionnaire. *M-Living together/cohabiting - married and living together/ not married but cohabiting, *M-Not living together/not cohabiting - married but not living together, not married and not cohabiting, *Substance use - combination of alcohol, cigarette and drug use 30 days prior enrolment.
