# Supplementary material for: Neurodevelopment of HIV-exposed uninfected children in Cape Town, South Africa
Source: PLoS One. 2020 Nov 18;15(11):e0242244. doi: 10.1371/journal.pone.0242244 (PMC7673492; doi:10.1371/journal.pone.0242244)
Supplement: S2 Table — (PDF) [file pone.0242244.s002.pdf]

S2 Table. Maternal factors associated with LTFU (n = 505)

		Loss-to-follow-up (LTFU)			
**Characteristics**	**N (%)**	**OR (95% CI)**	**p-value**	**aOR (95% CI)**	**p-value**
At baseline					
Age (years)					
<24	44 (12)	1.00 (Ref)		1.00 (Ref)	
25-29	100 (28)	**0.53 (0.28-0.98)**	**0.044**	0.55 (0.29-1.07)	0.078
30-34	123 (35)	**0.37 (0.20-0.71)**	**0.003**	**0.46 (0.23-0.92)**	**0.029**
≥35	88 (25)	**0.26 (0.12-0.57)**	**0.001**	0.48 (0.21-1.10)	0.084
BMI (kg/m^2)					
Normal (18.5-24.9)	88 (25)	1.00 (Ref)		1.00 (Ref)	
Underweight (<18.5)	6 (2)	2.45 (0.65-9.19)	0.184	3.05 (0.81-11.44)	0.098
Overweight (25-29.9)	90 (25)	**0.33 (0.15-0.72)**	**0.006**	**0.40 (0.18-0.88)**	**0.023**
Obese (≥30)	156 (44)	0.74 (0.44-1.24)	0.250	0.86 (0.49-1.52)	0.612
Relationship Status					
*M-Not living together/not cohabiting	167 (47)	1.00 (Ref)			
*M-Living together/cohabiting	176 (50)	0.90 (0.56-1.45)	0.671		
Not in a relationship	9 (3)	1.09 (0.30-3.99)	0.899		
SES					
Middle	108 (30)	1.00 (Ref)		1.00 (Ref)	
Lower	107 (30)	1.09 (0.60-2.00)	0.777	1.24 (0.65-2.37)	0.518
Higher	135 (38)	1.30 (0.74-2.30)	0.361	1.48 (0.81-2.73)	0.204
*Substance use					
No	271 (76)	1.00 (ref)			
Yes	79 (22)	1.16 (0.67-2.02)	0.598		
Parity					
Nulliparous	73 (21)	1.00 (Ref)			
Multiparous	279 (79)	0.65 (0.39-1.09)	0.102		
ART initiation status					
During pregnancy	174 (49)	1.00 (Ref)		1.00 (Ref)	
Pre-pregnancy	181 (51)	**0.26 (0.15-0.45)**	**<0.001**	**0.29 (0.17-0.51)**	**<0.001**

BMI - body mass index, SES - socioeconomic status, ART - antiretroviral therapy, OR - odds ratio. *M-Living together/Cohabiting - married and living together/ not married but cohabiting, *M-Not living together/not cohabiting - married but not living together, not married and not cohabiting, *Substance use - combination of alcohol, cigarette and drug use 30 days prior enrolment. Missing data for n = 505, n (%): BMI n=30 (5.9), Relationship status n=4 (0.8), SES n=9 (1.8), Substance use n=6 (1.2), Parity n=8 (1.6). Where data are missing on predictors, cases were included in the reference category in the regression. Interpretation of OR's: Predictor was associated with increased (OR>1) or decreases (OR<1) odds of having delayed (domain name) neurodevelopment compared to reference category (for that predictor).
