# Supplementary material for: Neurodevelopment of HIV-exposed uninfected children in Cape Town, South Africa
Source: PLoS One. 2020 Nov 18;15(11):e0242244. doi: 10.1371/journal.pone.0242244 (PMC7673492; doi:10.1371/journal.pone.0242244)
Supplement: S3 Table — (PDF) [file pone.0242244.s003.pdf]

S3 Table. Associations between maternal, child factors and delayed neurodevelopment on combined ASQ domains adjusted for maternal and child factors in one model (n = 355)

		ASQ Neurodevelopment Domains (Reference category – No delay)			
	**Total**	**Gross + Fine motor**		**Comm + ProbSolv + PerSocial**	
**Characteristics**	**N (%)**	**aOR (95% CI)**	**p-value**	**aOR (95% CI)**	**p-value**
**Maternal**					
At baseline					
Age (years)					
<24	44 (12)	1.00 (Ref)		1.00 (Ref)	
25-29	100 (28)	1.27 (0.55-2.94)	0.575	0.79 (0.26-2.37)	0.671
30-34	123 (35)	0.86 (0.36-2.03)	0.731	0.59 (0.20-1.71)	0.329
≥35	88 (25)	0.94 (0.39-2.29)	0.891	**0.22 (0.05-0.91)**	**0.036**
BMI (kg/m^2)					
Normal (18.5-24.9)	88 (25)	1.00 (Ref)		1.00 (Ref)	
Underweight (<18.5)	6 (2)	2.39 (0.36-15.81)	0.364	**6.72 (1.05-43.00)**	**0.044**
Overweight (25-29.9)	90 (25)	0.83 (0.42-1.64)	0.599	2.04 (0.83-5.04)	0.122
Obese (≥30)	156 (44)	0.69 (0.37-1.29)	0.247	1.06 (0.44-2.56)	0.894
SES					
Middle	108 (30)	1.00 (Ref)		1.00 (Ref)	
Lower	107 (30)	1.07 (0.57-2.02)	0.831	0.90 (0.35-2.36)	0.837
Higher	135 (38)	**0.40 (0.21-0.77)**	**0.006**	0.76 (0.31-1.85)	0.546
ART initiation status					
During pregnancy	174 (49)	1.00 (Ref)		1.00 (Ref)	
Pre-pregnancy	181 (51)	1.36 (0.78-2.37)	0.278	1.75 (0.85-3.63)	0.130
**Child**					
At birth					
Gender					
Male	199 (56)	1.00 (Ref)		1.00 (Ref)	
Female	156 (44)	0.77 (0.45-1.31)	0.335	0.56 (0.26-1.21)	0.139
Size for GA (percentile)					
Appropriate (10-90th)	270 (76)	1.00 (Ref)		1.00 (Ref)	
Small (<10th)	56 (16)	0.70 (0.32-1.51)	0.359	1.52 (0.63-3.68)	0.349
Large (>90th)	28 (8)	0.77 (0.27-2.19)	0.619	0.57 (0.13-2.53)	0.457
Gestation at delivery (weeks)					
Term delivery (≥37)	272 (77)	1.00 (Ref)		1.00 (Ref)	
Spontaneous preterm (<37)	22 (6)	1.45 (0.60-3.53)	0.412	1.75 (0.54-5.66)	0.349
Medically-indicated preterm (<37)	29 (8)	1.19 (0.48-2.94)	0.701	0.57 (0.13-2.53)	0.457
Between birth and assessment					
Breastfeeding duration					
Never	22 (6)	1.00 (Ref)		1.00 (Ref)	
Ever	319 (90)	1.08 (0.42-2.80)	0.869	0.90 (0.29-2.78)	0.849
At assessment					
Age (months)	355 (100)	0.96 (0.87-1.06)	0.411	1.01 (0.89-1.56)	0.849
Weight-for-age (g)	355 (100)	0.92 (0.76-1.10)	0.353	1.12 (0.91-1.38)	0.287

BMI - body mass index, SES - socioeconomic status, ART - antiretroviral therapy, GA - gestational age, ASQ - Ages & Stages Questionnaire, OR - odds ratio. Gross + Fine motor: combined gross motor & fine motor domains; Comm + ProbSolv + PerSocial: combined communication & problem solving & personal social domains. Missing data for n = 355, n (%): BMI n=15 (4.2), SES n=5 (1.4), Size for GA and Breastfeeding n=1 (0.3). Where data are missing on predictors, cases were included in the reference category in the regression. Interpretation of OR's for categorical predictors: Predictor was associated with increased (OR>1) or decreases (OR<1) odds of having delayed (domain name) neurodevelopment compared to reference category (for that predictor). Interpretation of OR's for continuous predictors: Unit increase in predictor was associated with increased (OR>1) or decreases (OR<1) odds of having delayed (domain name) neurodevelopment.
