# Supplementary material for: Neurodevelopment of HIV-exposed uninfected children in Cape Town, South Africa
Source: PLoS One. 2020 Nov 18;15(11):e0242244. doi: 10.1371/journal.pone.0242244 (PMC7673492; doi:10.1371/journal.pone.0242244)
Supplement: S4 Table — (PDF) [file pone.0242244.s004.pdf]

S4 Table. Frequencies of individual ASQ neurodevelopment domains for SGA children stratified by maternal ART initiation status (n = 56)

		**ART initiation status**		
**Neurodevelopment Sub-scale**	**Total** N (%) (n = 56)	**Pre-pregnancy** N (%) (n = 33)	**During pregnancy** N (%) (n = 23)	**p- value**
Gross motor				0.710
No delay	46 (82)	26 (79)	20 (87)	
Intermediate	4 (7)	3 (9)	1 (4)	
Delay	6 (11)	4 (12)	2 (8)	
Median (IQR)	55 (45-60)	55 (45-60)	55 (45-60)	
Fine motor				0.931
No delay	47 (84)	28 (85)	19 (83)	
Intermediate	4 (7)	2 (6)	2 (8)	
Delay	5 (9)	3 (9)	2 (8)	
Median (IQR)	50 (48-55)	50 (50-55)	50 (45-50)	
Communication				0.669
No delay	52 (93)	52 (93)	22 (96)	
Intermediate	1 (2)	1 (3	0	
Delay	3 (5)	2 (6)	1 (4)	
Median (IQR)	55 (45-60)	55 (45-60)	55 (45-60)	
Problem-solving				0.920
No delay	50 (89)	29 (88)	21 (91)	
Intermediate	3 (5)	2 (6)	1 (4)	
Delay	3 (5)	2 (6)	1 (4)	
Median (IQR)	55 (50-60)	55 (50-60)	55 (50-60)	
Personal-social				0.153
No delay	48 (86)	29 (88)	19 (83)	
Intermediate	2 (4)	0	2 (9)	
Delay	5 (9)	4 (12)	1 (4)	
Median (IQR)	50 (43-60)	50 (45-55)	50 (35-60)	
