# Supplementary material for: Neurodevelopment of HIV-exposed uninfected children in Cape Town, South Africa
Source: PLoS One. 2020 Nov 18;15(11):e0242244. doi: 10.1371/journal.pone.0242244 (PMC7673492; doi:10.1371/journal.pone.0242244)
Supplement: S5 Table — (PDF) [file pone.0242244.s005.pdf]

S5 Table. Unadjusted associations between maternal factors and delayed neurodevelopment on combined ASQ domains for SGA children (n = 56)

		ASQ Neurodevelopment Domains (Reference category – No delay) Unadjusted OR's			
	Total	Gross + Fine motor		Comm + ProbSolv + PerSocial	
**Characteristics**	**N (%)**	**OR (95% CI)**	**p-value**	**OR (95% CI)**	**p-value**
At baseline					
Age (years)					
<24	10 (18)	1.00 (ref)		1.00 (ref)	
25-29	14 (25)	0.64 (0.10-4.16)	0.637	1.09 (0.14-8.27)	0.933
30-34	14 (25)	0.93 (0.15-5.63)	0.940	1.09 (0.14-8.27)	0.933
≥35	18 (32)	0.67 (0.11-3.90)	0.653	0.24 (0.02-3.06)	0.269
BMI (kg/m^2)					
Normal (18.5-24.9)	23 (41)	1.00 (ref)		1.00 (ref)	
Underweight (<18.5)	2 (4)	2.29 (0.12-43.11)	0.581	4.75 (0.23-96.98)	0.311
Overweight (25-29.9)	11 (20)	0.86 (0.17-4.29)	0.851	2.71 (0.52-14.24)	0.238
Obese (≥30)	20 (36)	0.40 (0.09-1.86)	0.244	-----	
Relationship Status					
*M-Not living together/not cohabiting	31 (55)	1.00 (ref)		1.00 (ref)	
*M-Living together/cohabiting	23 (41)	0.82 (0.51-6.49)	0.354	1.09 (0.56-4.69)	0.903
No relationship	2 (4)	4.17 (0.22-78.65)	0.341	------	
SES					
Middle	18 (32)	1.00 (ref)		1.00 (ref)	
Lower	19 (34)	0.38 (0.08-1.84)	0.226	0.94 (0.16-5.47)	0.943
Higher	19 (34)	0.71 (0.17-2.98)	0.644	0.94 (0.16-5.47)	0.943
*Substance use					
No	48 (86)	1.00 (ref)		1.00 (ref)	
Yes	8 (14)	0.38 (0.04-3.50)	0.397	0.71 (0.08-6.77)	0.769
Parity					
Nulliparous	17 (30)	1.00 (ref)		1.00 (ref)	
Multiparous	39 (70)	0.72 (0.20-2.62)	0.619	0.48 (0.11-2.09)	0.327
ART initiation status					
During pregnancy	23 (41)	1.00 (ref)		1.00 (ref)	
Pre-pregnancy	33 (59)	1.35 (0.38-4.78)	0.642	1.48 (0.33-6.74)	0.611
At child's assessment					
ART Adherence					
Adherent	48 (86)	1.00 (ref)		1.00 (ref)	
Default	8 (14)	1.00 (0.17-5.72)	1.00	0.71 (0.08-6.77)	0.769

BMI - body mass index, SES - socioeconomic status, ART - antiretroviral therapy, ASQ - Ages & Stages Questionnaire, OR - odds ratio. *M-Living together/cohabiting - married and living together/ not married but cohabiting, *M-Not living together/not cohabiting - married but not living together, not married and not cohabiting, *Substance use - combination of alcohol, cigarette and drug use 30 days prior enrolment. Gross + Fine motor: combined gross motor & fine motor domains; Comm + ProbSolv + PerSocial: combined communication & problem solving & personal social domains. Missing data for n = 56, n (%): BMI and SES and Substance use n=2 (3.6), Relationship status n=1 (1.8). Where data are missing on predictors, cases were included in the reference category in the regression. Interpretation of OR's: Predictor was associated with increased (OR>1) or decreases (OR<1) odds of having delayed (domain name) neurodevelopment compared to reference category (for that predictor).
