# Supplementary material for: Neurodevelopment of HIV-exposed uninfected children in Cape Town, South Africa
Source: PLoS One. 2020 Nov 18;15(11):e0242244. doi: 10.1371/journal.pone.0242244 (PMC7673492; doi:10.1371/journal.pone.0242244)
Supplement: S6 Table — (PDF) [file pone.0242244.s006.pdf]

S6 Table. Unadjusted associations between maternal factors and delayed neurodevelopment on individual ASQ domains for SGA children (n = 56)

		ASQ Neurodevelopment Domains (Reference category – No delay)									
	**Total**	**Gross motor**		**Fine motor**		**Communication**		**Problem-solving**		**Personal-social**	
**Characteristics**	**N (%)**	**OR (95% CI)**	**p-value**	**OR (95% CI)**	**p-value**	**OR (95% CI)**	**p-value**	**OR (95% CI)**	**p-value**	**OR (95% CI)**	**p-value**
At baseline											
Age (years)											
<24	10 (18)	1.00 (Ref)		1.00 (Ref)		1.00 (Ref)		1.00 (Ref)		1.00 (Ref)	
25-29	14 (25)	0.67 (0.08-5.86)	0.715	0.39 (0.05-2.98)	0.363	1.50 (0.11-19.91)	0.759	0.31 (0.02-4.06)	0.371	0.67 (0.08-5.86)	0.715
30-34	14 (25)	1.09 (0.14-8.27)	0.933	0.39 (0.05-2.98)	0.363	0.69 (0.04-13.07)	0.806	0.67 (0.08-5.86)	0.715	0.73 (0.08-6.44)	0.775
≥35	18 (32)	0.80 (0.11-5.92)	0.827	0.29 (0.04-2.19)	0.231	-------		0.24 (0.02-3.06)	0.269	0.24 (0.02-3.06)	0.269
BMI (kg/m^2)											
Normal (18.5-24.9)	23 (41)	1.00 (Ref)		1.00 (Ref)		1.00 (Ref)		1.00 (Ref)		1.00 (Ref)	
Underweight (<18.5)	2 (4)	3.60 (0.18-70.19)	0.398	2.83 (0.15-	0.489	10.50 (0.44-250.66)	0.146	6.67 (0.31-143.43)		4.50 (0.22-92.16)	0.329
Overweight (25-29.9)	11 (20)	0.80 (0.13-5.04)	0.812	54.15)	0.278	1.05 (0.08-13.47)	0.970	1.48 (0.20-10.75)		1.00 (0.15-6.71)	1.000
Obese (≥30)	20 (36)	0.40 (0.07-2.37)	0.313	0.28 (0.03-2.76)	0.095	-------		-------		-------	
				0.15 (0.02-1.39)							
Relationship Status											
*M-Not living together/not cohabiting	31 (55)	1.00 (Ref)		1.00 (Ref)		1.00 (Ref)		1.00 (Ref)		1.00 (Ref)	
*M-Living together/cohabiting	23 (41)	3.29 (0.72-15.14)	0.125	0.63 (0.14-2.86)	0.544	1.38 (0.18-10.81	0.759	0.64 (0.11-3.92)	0.632	0.52 (0.09-3.01)	0.466
No relationship	2 (4)	9.33 (0.44-195.90)	0.150	-------		-------		-------		-------	
SES											
Middle	18 (32)	1.00 (Ref)		1.00 (Ref)		1.00 (Ref)		1.00 (Ref)		1.00 (Ref)	
Lower	19 (34)	0.31 (0.05-1.87)	0.199	0.19 (0.02-1.98)	0.167	0.44 (0.04-5.50)	0.528	0.28 (0.03-3.02)	0.293	0.88 (0.11-7.19)	0.907
Higher	19 (34)	0.49 (0.10-2.47)	0.385	0.93 (0.19-4.53)	0.932	0.44 (0.04-5.50)	0.528	0.59 (0.08-4.08)	0.591	1.41 (0.20-9.79)	0.731
*Substance use											
No	48 (86)	1.00 (Ref)		1.00 (Ref)		1.00 (Ref)		1.00 (Ref)		1.00 (Ref)	
Yes	8 (14)	0.62 (0.07-5.80)	0.674	-------		-------		1.23 (0.12-12.40)	0.861	-------	
Parity											
Nulliparous	17 (30)	1.00 (Ref)		1.00 (Ref)		1.00 (Ref)		1.00 (Ref)		1.00 (Ref)	
Multiparous	39 (70)	1.02 (0.23-4.60)	0.979	0.48 (0.11-2.09)	0.327	0.41 (0.05-3.21)	0.392	0.86 (0.14-5.28)	0.868	0.55 (0.11-2.82)	0.473
ART initiation status											
During pregnancy	23 (41)	1.00 (Ref)		1.00 (Ref)		1.00 (Ref)		1.00 (Ref)		1.00 (Ref)	
Pre-pregnancy	33 (59)	1.79 (0.41-7.93)	0.440	0.85 (0.20-3.62)	0.824	2.20 (0.21-23.07)	0.511	1.45 (0.24-8.80)	0.687	0.87 (0.17-4.41)	
At child's assessment											
ART Adherence											
Adherent	48 (86)	1.00 (Ref)		1.00 (Ref)		1.00 (Ref)		1.00 (Ref)		1.00 (Ref)	
Default	8 (14)	1.67 (0.28-9.95)	0.575	0.71 (0.08-6.77)	0.769	2.14 (0.19-24.12)	0.537	1.23 (0.12-12.40)	0.861	0.98 (0.10-9.59)	0.984

BMI - body mass index, SES - socioeconomic status, ART - antiretroviral therapy, GA - gestational age, ASQ - Ages & Stages Questionnaire, OR - odds ratio. *M-Living together/cohabiting - married and living together/ not married but cohabiting, *M-Not living together/not cohabiting - married but not living together, not married and not cohabiting, *Substance use - combination of alcohol, cigarette and drug use 30 days prior enrolment. Missing data for n = 56, n (%): BMI and SES and Substance use n=2 (3.6), Relationship status n=1 (1.8). Where data are missing on predictors, cases were included in the reference category in the regression. Interpretation of OR's: Predictor was associated with increased (OR>1) or decreases (OR<1) odds of having delayed (domain name) neurodevelopment compared to reference category (for that predictor).
