# Supplementary material for: Neurodevelopment of HIV-exposed uninfected children in Cape Town, South Africa
Source: PLoS One. 2020 Nov 18;15(11):e0242244. doi: 10.1371/journal.pone.0242244 (PMC7673492; doi:10.1371/journal.pone.0242244)
Supplement: S7 Table — (PDF) [file pone.0242244.s007.pdf]

S7 Table. Unadjusted associations between maternal, child factors and delayed neurodevelopment on individual ASQ domains (n = 355)

Characteristics	Total N (%)	Gross motor OR (95% CI)	Gross motor p-value	Fine motor OR (95% CI)	Fine motor p-value	Communication OR (95% CI)	Communication p-value	Problem-solving OR (95% CI)	Problem-solving p-value	Personal-social OR (95% CI)	Personal-social p-value
		ASQ Neurodevelopment Domains (Reference category – No delay)									
**Maternal**											
At baseline											
Age (years)											
<24	44 (12)	1.00 (Ref)		1.00 (Ref)		1.00 (Ref)		1.00 (Ref)		1.00 (Ref)	
25-29	100 (28)	2.05 (0.65-6.50)	0.223	0.70 (0.30-1.64)	0.416	0.75 (0.21-2.72)	0.665	0.87 (0.21-3.67)	0.852	0.88 (0.28-2.74)	0.820
30-34	123 (35)	1.50 (0.47-4.75)	0.495	0.80 (0.36-1.81)	0.597	0.78 (0.23-2.73)	0.718	0.95 (0.24-3.76)	0.943	0.79 (0.26-2.41)	0.675
≥35	88 (25)	2.05 (0.64-6.62)	0.228	0.72 (0.30-1.71)	0.453	0.35 (0.08-1.66)	0.187	0.48 (0.09-2.50)	0.385	0.57 (0.16-2.00)	0.379
BMI (kg/m^2)											
Normal (18.5-24.9)	88 (25)	1.00 (Ref)		1.00 (Ref)		1.00 (Ref)		1.00 (Ref)		1.00 (Ref)	
Underweight (<18.5)	6 (2)	1..21 (0.38-13.00)	0.380	3.68 (0.69-19.57)	0.126	6.86 (1.06-44.29)	0.043	3.92 (0.38-40.32)	0.251	2.04 (0.21-19.53)	0.535
Overweight (25-29.9)	90 (25)	0.96 (0.46-2.00)	0.904	0.92 (0.46-1.85)	0.817	0.82 (0.25-2.67)	0.737	1.65 (0.50-5.41)	0.406	1.44 (0.57-3.66)	0.442
Obese (≥30)	156 (44)	**0.47 (0.23-0.98)**	**0.043**	0.88 (0.47-1.63)	0.676	0.84 (0.30-2.33)	0.738	0.92 (0.28-2.99)	0.891	0.78 (0.31-1.96)	0.598
Relationship Status											
*M-Not living together/not cohabiting	167 (47)	1.00 (Ref)		1.00 (Ref)		1.00 (Ref)		1.00 (Ref)		1.00 (Ref)	
*M-Living together/cohabiting	176 (50)	1.11 (0.61-2.03)	0.729	1.13 (0.67-1.91)	0.643	0.84 (0.36-1.96)	0.688	1.15 (0.46-2.85)	0.764	0.93 (0.44-1.96)	0.841
Not relationship	9 (3)	1.79 (0.12-3.16)	0.486	0.51 (0.06-4.21)	0.530	------		------		2.86 (0.54-15.04)	0.215
SES											
Middle	108 (30)	1.00 (Ref)		1.00 (Ref)		1.00 (Ref)		1.00 (Ref)		1.00 (Ref)	
Lower	107 (30)	0.89 (0.44-1.77)	0.733	0.97 (0.53-1.80)	0.934	0.91 (0.32-2.61)	0.861	0.40 (0.12-1.32)	0.132	0.73 (0.28-1.91)	0.526
Higher	135 (38)	**0.47 (0.22-0.98)**	**0.045**	**0.50 (0.26-0.95)**	**0.034**	0.82 (0.30-2.26)	0.700	0.48 (0.17-1.36)	0.168	0.98 (0.42-2.30)	0.972
*Substance use											
No	271 (76)	1.00 (Ref)		1.00 (Ref)		1.00 (Ref)		1.00 (Ref)		1.00 (Ref)	
Yes	79 (22)	0.81 (0.38-1.69)	0.572	0.63 (0.32-1.24)	0.184	1.25 (0.47-3.28)	0.654	0.87 (0.28-2.67)	0.803	1.44 (0.64-3.27)	0.378
Parity											
Nulliparous	73 (21)	1.00 (Ref)		1.00 (Ref)		1.00 (Ref)		1.00 (Ref)		1.00 (Ref)	
Multiparous	279 (79)	1.02 (0.50-2.09)	0.961	1.49 (0.76-2.94)	0.248	0.60 (0.24-1.52)	0.284	1.58 (0.45-5.55)	0.476	0.97 (0.40-2.34)	0.942
ART initiation status											
During pregnancy	174 (49)	1.00 (Ref)		1.00 (Ref)		1.00 (Ref)		1.00 (Ref)		1.00 (Ref)	
Pre-pregnancy	181 (51)	1.37 (0.76-2.49)	0.297	0.98 (0.59-1.65)	0.954	0.88 (0.38-2.05)	0.765	0.96 (0.39-2.37)	0.928	**2.30 (1.05-5.02)**	**0.037**
At child's assessment											
ART Adherence											
Adherent	319 (90)	1.00 (Ref)		1.00 (Ref)		1.00 (Ref)		1.00 (Ref)		1.00 (Ref)	
Default	36 (10)	1.19 (0.47-3.01)	0.718	1.82 (0.85-3.91)		0.38 (0.05-2.95)	0.358	0.45 (0.06-3.48)	0.445	1.28 (0.42-3.89)	0.662
**Child**											
At birth											

Gender											
Male	199 (56)	1.00 (Ref)		1.00 (Ref)		1.00 (Ref)		1.00 (Ref)		1.00 (Ref)	
Female	156 (44)	0.63 (0.34-1.17)	0.145	0.75 (0.44-1.27)	0.282	0.53 (0.21-1.33)	0.180	0.67 (0.26-1.73)	0.410	**0.39 (0.17-0.91)**	**0.028**
Birthweight (g)											
Normal (2500-4000)	283 (80)	1.00 (Ref)		1.00 (Ref)		1.00 (Ref)		1.00 (Ref)		1.00 (Ref)	
Low (<2500)	57 (16)	1.86 (0.92-3.78)	0.084	0.70 (0.33-1.52)	0.369	0.47 (0.11-2.05)	0.321	2.28 (0.83-6.21)	0.108	1.54 (0.63-3.77)	0.349
High (>4000)	13 (4)	------		1.67 (0.50-5.61)	0.409	-----		-----		0.90 (0.11-7.21)	0.918
Size for GA (percentile)											
Appropriate (10-90th)	270 (76)	1.00 (Ref)		1.00 (Ref)		1.00 (Ref)		1.00 (Ref)		1.00 (Ref)	
Small (<10th)	56 (16)	1.26 (0.59-2.69)	0.559	0.67 (0.31-1.45)	0.314	1.02 (0.33-3.12)	0.978	2.38 (0.68-6.57)	0.094	1.48 (0.60-3.64)	0.390
Large (>90th)	28 (8)	0.44 (0.10-1.95)	0.282	0.59 (0.20-1.76)	0.340	------		0.74 (0.09-5.86)	0.771	0.38 (0.05-2.90)	0.349
Gestation at delivery (weeks)											
Term delivery (≥37)	272 (77)	1.00 (Ref)		1.00 (Ref)		1.00 (Ref)		1.00 (Ref)		1.00 (Ref)	
Spontaneous preterm (<37)	22 (6)	1.41 (0.45-4.41)	0.553	1.19 (0.42-3.37)	0.747	------		0.95 (0.12-7.63)	0.961	1.77 (0.48-6.45)	0.390
Medically-indicated preterm (<37)	29 (8)	1.32 (0.47-3.69)	0.593	0.65 (0.22-1.94)	0.435	------		1.48 (0.32-6.90)	0.621	0.40 (0.05-3.09)	0.379
Head circumference (cm)	317 (89)	1.01 (0.87-1.17)	0.868	1.00 (.89-1.13)	0.947	1.01 (0.95-1.08)	0.661	**0.73 (0.58-0.93)**	**0.009**	0.95 (0.82-1.10)	0.484
Length (cm)	312 (88)	0.94 (0.88-1.01)	0.081	0.98 (0.92-1.04)	0.596	0.98 (0.90-1.06)	0.595	0.89 (0.79-1.01)	0.064	0.98 (0.90-1.06)	0.530
Between birth and assessment											
Breastfeeding duration											
Never	22 (6)	1.00 (Ref)		1.00 (Ref)		1.00 (Ref)		1.00 (Ref)		1.00 (Ref)	
Ever	319 (90)	0.84 (0.33-2.14)	0.718	0.75 (0.34-1.68)	0.489	0.37 (0.13-1.07)	0.067	2.22 (0.29-17.12)	0.445	1.12 (0.32-3.86)	0.863
<6 months	178 (50)	1.00 (Ref)		1.00 (Ref)		1.00 (Ref)		1.00 (Ref)		1.00 (Ref)	
≥6 months	163 (46)	0.63 (0.34-1.16)	0.136	0.67 (0.39-1.15)	0.145	0.80 (0.33-1.96)	0.631	0.45 (0.17-1.20)	0.109	0.50 (0.23-1.10)	0.086
Hospital admission											
No	295 (83)	1.00 (Ref)		1.00 (Ref)		1.00 (Ref)		1.00 (Ref)		1.00 (Ref)	
Yes	60 (17)	**2.32 (1.18-4.58)**	**0.015**	0.84 (0.41-1.72)	0.640	1.81 (0.68-4.81)	0.234	**2.87 (1.09-7.53)**	**0.033**	2.11 (0.92-4.82)	0.078
Missed vaccinations											
No	217 (61)	1.00 (Ref)		1.00 (Ref)		1.00 (Ref)		1.00 (Ref)		1.00 (Ref)	
Yes	138 (39)	1.30 (0.71-2.35)	0.392	1.05 (0.62-1.77)	0.867	1.23 (0.52-2.87)	0.648	1.31 (0.53-3.24)	0.564	1.24 (0.59-2.58)	0.567
At assessment											
Age (months)	355 (100)	0.94 (0.85-1.05)	0.260	1.01 (0.93-1.09)	0.835	1.12 (1.00-1.25)	0.053	1.05 (0.93-1.18)	0.443	0.97 (0.86-1.10)	0.671
Weight (kg)	355 (100)	1.00 (1.00-1.01)	0.500	1.00 (1.00-1.01)	0.628	1.00 (1.00-1.01)	0.356	1.00 (1.00-1.01)	0.923	1.00 (1.00-1.01)	0.795
Height (cm)	353 (99)	0.96 (0.90-1.01)	0.139	0.98 (0.93-1.03)	0.418	1.10 (1.00-1.21)	0.046	1.00 (0.91-1.09)	0.925	0.99 (0.93-1.05)	0.637
MUAC (cm)	354 (99)	0.93 (0.76-1.14)	0.504	0.93 (0.80-1.08)	0.353	0.98 (0.76-1.26)	0.846	1.02 (0.77-1.36)	0.872	1.08 (0.88-1.33)	0.441

Head circumference (cm)	354 (99)	0.94 (0.78-1.14)	0.562	0.94 (0.82-1.08)	0.374	0.99 (0.74-1.33)	0.945	0.89 (0.68-1.17)	0.390	0.98 (0.81-1.18)	0.801
Weight-for-age (g)	355 (100)	0.93 (0.74-1.17)	0.546	0.94 (0.79-1.13)	0.512	1.01 (0.78-1.31)	0.924	0.97 (0.69-1.36)	0.854	1.03 (0.82-1.29)	0.779
Height-for-age (cm)	353 (99)	0.91 (0.76-1.09)	0.292	0.90 (0.76-1.07)	0.227	1.05 (0.80-1.38)	0.741	0.89 (0.72-1.11)	0.315	0.91 (0.76-1.10)	0.340
Weight-for-height	352 (99)	0.99 (0.80-1.22)	0.910	1.00 (0.85-1.18)	0.970	0.99 (0.83-1.19)	0.931	1.03 (0.82-1.30)	0.799	1.09 (0.92-1.28)	0.330

BMI - body mass index, SES - socioeconomic status, ART - antiretroviral therapy, GA - gestational age, MUAC - mid-upper arm circumference, ASQ - Ages & Stages Questionnaire, OR - odds ratio. *M-Living together/cohabiting - married and living together/ not married but cohabiting, *M-Not living together/not cohabiting - married but not living together, not married and not cohabiting, *Substance use - combination of alcohol, cigarette and drug use 30 days prior enrolment. Missing data for n = 355, n (%): BMI n=15 (4.2), Relationship status n=3 (0.9), SES and Substance use n=5 (1.4), Parity and ART adherence at 12 months n=3 (0.9), Birthweight, Height at 12 months and Height-for-age n=2 (0.6), Size for GA, Breastfeeding and Head circumference at 12 months n=1 (0.3), Birth head circumference n=38 (10.7), Birth length n=43 (12.1), Weight-for-height n = 3 (0.8), ASQ version n=7 (2.0). Where data are missing on predictors, cases were included in the reference category in the regression. Interpretation of OR's for categorical predictors: Predictor was associated with increased (OR>1) or decreases (OR<1) odds of having delayed (domain name) neurodevelopment compared to reference category (for that predictor). Interpretation of OR's for continuous predictors: Unit increase in predictor was associated with increased (OR>1) or decreases (OR<1) odds of having delayed (domain name) neurodevelopment.
