# Supplementary material for: Neurodevelopment of HIV-exposed uninfected children in Cape Town, South Africa
Source: PLoS One. 2020 Nov 18;15(11):e0242244. doi: 10.1371/journal.pone.0242244 (PMC7673492; doi:10.1371/journal.pone.0242244)
Supplement: S8 Table — (PDF) [file pone.0242244.s008.pdf]

S8 Table. Associations between maternal, child factors and delayed neurodevelopment on individual ASQ domains adjusted for maternal and child factors on two separatemodels (n = 355)

		ASQ Neurodevelopment Domains (Reference category – No delay)									
	**Total**	**Gross motor**		**Fine motor**		**Communication**		**Problem-solving**		**Personal-social**	
**Characteristics**	**N (%)**	**aOR (95% CI)**	**p-value**	**aOR (95% CI)**	**p-value**	**aOR (95% CI)**	**p-value**	**aOR (95% CI)**	**p-value**	**aOR (95% CI)**	**p-value**
**Maternal**											
At baseline											
Age (years)											
<24	44 (12)	1.00 (Ref)		1.00 (Ref)		1.00 (Ref)	0.639	1.00 (Ref)		1.00 (Ref)	
25-29	100 (28)	2.45 (0.79-7.56)	0.119	0.72 (0.31-1.67)	0.439	0.74 (0.21-2.61)	0.545	0.92 (0.21-3.92)	0.905	0.99 (0.30-3.27)	0.991
30-34	123 (35)	1.59 (0.51-4.96)	0.420	0.77 (0.34-1.71)	0.514	0.70 (0.22-2.25)	0.156	0.91 (0.23-3.63)	0.890	0.75 (0.24-2.39)	0.631
≥35	88 (25)	2.23 (0.69-7.25)	0.181	0.69 (0.29-1.65)	0.400	0.34 (0.07-1.51)		0.51 (0.09-2.84)	0.445	0.47 (0.13-1.78)	0.269
BMI (kg/m^2)											
Normal (18.5-24.9)	88 (25)	1.00 (Ref)		1.00 (Ref)		1.00 (Ref)		1.00 (Ref)		1.00 (Ref)	
Underweight (<18.5)	6 (2)	2.43 (0.39-15.02)	0.338	4.37 (0.84-22.85)	0.080	**8.13 (1.26-52.38)**	**0.027**	6.01 (0.55-65.20)	0.140	2.04 (0.23-17.79)	0.519
Overweight (25-29.9)	90 (25)	0.87 (0.41-1.84)	0.720	0.94 (0.46-1.90)	0.861	0.88 (0.27-2.88)	0.828	1.74 (0.51-5.94)	0.379	1.34 (0.52-3.46)	0.546
Obese (≥30)	156 (44)	**0.42 (0.20-0.88)**	**0.021**	0.91 (0.49-1.69)	0.766	0.96 (0.37-2.52)	0.938	1.10 (0.33-3.68)	0.872	0.81 (0.32-2.07)	0.656
SES											
Middle	108 (30)	1.00 (Ref)		1.00 (Ref)		1.00 (Ref)		1.00 (Ref)		1.00 (Ref)	
Lower	107 (30)	0.86 (0.42-1.78)	0.689	0.97 (0.51-1.82)	0.916	0.88 (0.30-2.57)	0.811	0.39 (0.10-1.57)	0.184	0.71 (0.25-2.01)	0.518
Higher	135 (38)	**0.44 (0.21-0.92)**	**0.029**	**0.48 (0.25-0.91)**	**0.025**	0.75 (0.26-2.12)	0.585	0.45 (0.16-1.28)	0.135	0.91 (0.38-2.16)	0.823
ART initiation status											
During pregnancy	174 (49)	1.00 (Ref)		1.00 (Ref)		1.00 (Ref)		1.00 (Ref)		1.00 (Ref)	
Pre-pregnancy	181 (51)	1.41 (0.76-2.62)	0.275	1.01 (0.58-1.76)	0.960	1.02 (0.45-2.32)	0.964	1.10 (0.43-2.81)	0.838	**2.72 (0.38-2.16)**	**0.013**
**Child**											
At birth											
Gender											
Male	199 (56)	1.00 (Ref)		1.00 (Ref)		1.00 (Ref)		1.00 (Ref)		1.00 (Ref)	
Female	156 (44)	0.62 (0.32-1.20)	0.157	0.72 (0.40-1.28)	0.259	0.90 (0.33-2.50)	0.842	0.71 (0.25-2.05)	0.527	0.47 (0.20-1.13)	0.090
Size for GA (percentile)											
Appropriate (10-90th)	270 (76)	1.00 (Ref)		1.00 (Ref)		1.00 (Ref)		1.00 (Ref)		1.00 (Ref)	
Small (<10th)	56 (16)	1.09 (0.45-2.62)	0.852	0.60 (0.25-1.46)	0.261	1.06 (0.29-3.34)	0.932	2.50 (0.82-7.66)	0.109	1.70 (0.64-4.49)	0.284
Large (>90th)	28 (8)	0.43 (0.09-2.00)	0.282	0.62 (0.20-1.96)	0.418	-------		0.83 (0.10-6.99)	0.863	0.42 (0.05-3.32)	0.409
Gestation at delivery (weeks)											
Term delivery (≥37)	272 (77)	1.00 (Ref)		1.00 (Ref)		1.00 (Ref)		1.00 (Ref)		1.00 (Ref)	
Spontaneous preterm (<37)	22 (6)	1.28 (0.40-4.07)	0.674	1.01 (0.36-2.84)	0.986	-------		0.99 (0.12-8.17)	0.995	1.68 (0.45-6.27)	0.437
Medically-indicated preterm (<37)	29 (8)	1.49 (0.53-4.23)	0.450	0.68 (0.21-2.16)	0.511	-------		1.36 (0.28-6.61)	0.700	0.36 (0.05-2.76)	0.326

Between birth and assessment											
Breastfeeding duration											
Never	22 (6)	1.00 (Ref)		1.00 (Ref)		1.00 (Ref)		1.00 (Ref)		1.00 (Ref)	
Ever	319 (90)	0.80 (0.28-2.33)	0.684	1.03 (0.39-2.72)	0.945	1.22 (0.25-4.94)	0.889	------		1.66 (0.35-7.93)	0.523
At assessment											
Age (months)	355 (100)	0.92 (0.82-1.04)	0.174	0.97 (0.88-1.07)	0.572	**1.19 (1.05-1.34)**	**0.005**	1.08 (0.94-1.24)	0.297	0.95 (0.82-1.11)	0.525
Weight-for-age (g)	355 (100)	0.91 (0.71-1.18)	0.471	0.91 (0.73-1.12)	0.373	0.82 (0.54-1.24)	0.344	1.04 (0.76-1.41)	0.815	1.18 (0.95-1.47)	0.138

BMI - body mass index, SES - socioeconomic status, ART - antiretroviral therapy, GA - gestational age, ASQ - Ages & Stages Questionnaire, OR - odds ratio. Maternal model adjusted for age, BMI, SES and ART initiation status. Infant model adjusted for gender, size for GA, delivery GA, breastfeeding duration and weight-for-age at 12 months. Missing data for n = 355, n (%): BMI n=15 (4.2), SES n=5 (1.4), Size for GA and Breastfeeding n=1 (0.3). Where data are missing on predictors, cases were included in the reference category in the regression. Interpretation of OR's for categorical predictors: Predictor was associated with increased (OR>1) or decreases (OR<1) odds of having delayed (domain name) neurodevelopment compared to reference category (for that predictor). Interpretation of OR's for continuous predictors: Unit increase in predictor was associated with increased (OR>1) or decreases (OR<1) odds of having delayed (domain name) neurodevelopment.
