# Supplementary material for: Neurodevelopment of HIV-exposed uninfected children in Cape Town, South Africa
Source: PLoS One. 2020 Nov 18;15(11):e0242244. doi: 10.1371/journal.pone.0242244 (PMC7673492; doi:10.1371/journal.pone.0242244)
Supplement: S9 Table — (PDF) [file pone.0242244.s009.pdf]

S9 Table. Associations between maternal, child factors and delayed neurodevelopment on individual ASQ domains adjusted for maternal and child factors in one model (n =355)

		ASQ Neurodevelopment Domains (Reference category – No delay)									
	**Total**	**Gross motor**		**Fine motor**		**Communication**		**Problem-solving**		**Personal-social**	
**Characteristics**	**N (%)**	**aOR (95% CI)**	**p-value**	**aOR (95% CI)**	**p-value**	**aOR (95% CI)**	**p-value**	**aOR (95% CI)**	**p-value**	**aOR (95% CI)**	**p-value**
**Maternal**											
At baseline											
Age (years)	44 (12)	1.00 (Ref)		1.00 (Ref)		1.00 (Ref)		1.00 (Ref)		1.00 (Ref)	
<24	100 (28)	2.73 (0.80-9.38)	0.110	0.69 (0.28-1.70)	0.421	0.72 (015-3.55)	0.690	0.96 (0.18-5.14)	0.958	0.70 (0.20-2.50)	0.583
25-29	123 (35)	1.94 (0.56-6.71)	0.294	0.58 (0.24-1.42)	0.231	0.60 (0.16-2.33)	0.464	0.84 (0.17-4.07)	0.830	0.50 (0.13-1.87)	0.305
30-34	88 (25)	26.1 (0.76-9.31)	0.124	0.58 (0.22-1.50)	0.260	0.26 (0.04-1.90)	0.185	0.31 (0.04-2.19)	0.241	0.24 (0.05-1.21)	0.084
≥35											
BMI (kg/m²)											
Normal (18.5-24.9)	88 (25)	1.00 (Ref)		1.00 (Ref)		1.00 (Ref)		1.00 (Ref)		1.00 (Ref)	
Underweight (<18.5)	6 (2)	2.18 (0.29-16.25)	0.445	4.56 (0.75-27.54)	0.098	**10.43 (1.28-83.59)**	**0.028**	**16.81 (1.60-176.06)**	**0.019**	4.86 (0.56-42.50)	0.153
Overweight (25-29.9)	90 (25)	0.89 (0.40-1.99)	0.773	0.89 (0.42-1.88)	0.756	1.19 (0.32-4.44)	0.791	**7.78 (1.56-36.68)**	**0.012**	2.67 (0.89-8.00)	0.078
Obese (≥30)	156 (44)	**0.45 (0.20-0.99)**	**0.047**	0.84 (0.43-1.65)	0.613	0.78 (0.23-2.67)	0.696	3.83 (0.89-16.39)	0.070	1.48 (0.51-4.28)	0.467
SES											
Middle	108 (30)	1.00 (Ref)		1.00 (Ref)		1.00 (Ref)		1.00 (Ref)		1.00 (Ref)	
Lower	107 (30)	0.87 (0.39-1.91)	0.723	1.06 (0.53-2.11)	0.870	0.67 (0.17-2.73)	0.581	0.30 (0.07-1.28)	0.104	1.03 (0.32-3.33)	0.964
Higher	135 (38)	**0.43 (0.20-0.96)**	**0.040**	**0.43 (0.21-0.89)**	**0.024**	0.59 (0.17-2.11)	0.421	0.826 (0.07-1.00)	0.050	0.93 (0.31-2.84)	0.905
ART initiation status											
During pregnancy	174 (49)	1.00 (Ref)		1.00 (Ref)		1.00 (Ref)		1.00 (Ref)		1.00 (Ref)	
Pre-pregnancy	181 (51)	1.35 (0.70-2.61)	0.373	1.22 (0.66-2.29)	0.524	0.21 (0.41-3.55)	0.727	1.20 (0.39-3.68)	0.749	2.14 (0.87-5.30)	0.098
Gender											
Male	199 (56)	1.00 (Ref)		1.00 (Ref)		1.00 (Ref)		1.00 (Ref)		1.00 (Ref)	
Female	156 (44)	0.74 (0.38-1.43)	0.368	0.78 (0.44-1.41)	0.414	0.98 (0.33-2.89)	0.968	0.77 (0.25-2.35)	0.652	0.43 (0.17-1.08)	0.073
**Infant**											
At birth											
Size for GA (percentile)											
Appropriate (10-90th)	270 (76)	1.00 (Ref)		1.00 (Ref)		1.00 (Ref)		1.00 (Ref)		1.00 (Ref)	
Small (<10th)	56 (16)	1.09 (0.45-2.65)	0.853	0.54 (0.22-1.30)	0.168	1.12 (0.28-4.37)	0.875	**3.49 (1.14-10.62)**	**0.028**	1.82 (0.69-4.78)	0.224
Large (>90th)	28 (8)	0.46 (0.09-2.38)	0.355	0.68 (0.22-2.19)	0.527	-----		1.34 (0.13-14.36)	0.808	0.52 (0.07-3.66)	0.513
Gestation at delivery (weeks)											
Term delivery (≥37)	272 (77)	1.00 (Ref)		1.00 (Ref)		1.00 (Ref)		1.00 (Ref)		1.00 (Ref)	
Spontaneous preterm (<37)	22 (6)	1.23 (0.39-4.08)	0.701	1.02 (0.37-2.82)	0.965	------		1.05 (0.13-8.49)	0.965	1.68 (0.45-6.35)	0.442
Medically-indicated preterm (<37)	29 (8)	1.64 (0.56-4.82)	0.369	0.65 (0.19-2.20)	0.493	------		1.67 (0.35-8.06)	0.520	0.37 (0.06-2.48)	0.309

Between birth and assessment											
Breastfeeding duration											
Never	22 (6)	1.00 (Ref)		1.00 (Ref)		1.00 (Ref)		1.00 (Ref)		1.00 (Ref)	
Ever	319 (90)	0.74 (0.25-2.19)	0.590	0.89 (0.33-2.39)	0.823	0.82 (0.19-3.47)	0.783	------		1.62 (0.36-7.30)	0.532
At assessment											
Age (months)	355 (100)	0.95 (0.84-1.08)	0.431	0.98 (0.89-1.09)	0.763	**1.19 (1.03-1.37)**	**0.015**	1.13 (0.96-1.33)	0.138	0.97 (0.82-1.14)	0.695
Weight-for-age (g)	355 (100)	0.96 (0.73-1.25)	0.745	0.93 (0.74-1.16)	0.503	0.94 (0.63-1.40)	0.752	1.03 (0.75-1.42)	0.838	1.18 (0.93-1.50)	0.182

BMI - body mass index, SES - socioeconomic status, ART - antiretroviral therapy, GA - gestational age, ASQ - Ages & Stages Questionnaire, OR - odds ratio. Maternal model adjusted for age, BMI, SES and ART initiation status. Infant model adjusted for gender, size for GA, delivery GA, breastfeeding duration and weight-for-age at 12 months. Missing data for n = 355, n (%): BMI n=15 (4.2), SES n=5 (1.4), Size for GA and Breastfeeding n=1 (0.3). Where data are missing on predictors, cases were included in the reference category in the regression. Interpretation of OR's for categorical predictors: Predictor was associated with increased (OR>1) or decreases (OR<1) odds of having delayed (domain name) neurodevelopment compared to reference category (for that predictor). Interpretation of OR's for continuous predictors: Unit increase in predictor was associated with increased (OR>1) or decreases (OR<1) odds of having delayed (domain name) neurodevelopment.
